# Parental Birth-Related PTSD Symptoms and Bonding in the Early Postpartum Period: A Prospective Population-Based Cohort Study

**DOI:** 10.3389/fpsyt.2020.570727

**Published:** 2020-09-23

**Authors:** Suzannah Stuijfzand, Susan Garthus-Niegel, Antje Horsch

**Affiliations:** ^1^Faculty of Biology and Medicine, Institute of Higher Education and Research in Healthcare-IUFRS, University of Lausanne, Lausanne, Switzerland; ^2^Department of Medicine, Faculty of Human Sciences, Medical School Hamburg, Hamburg, Germany; ^3^Faculty of Medicine, Institute and Policlinic of Occupational and Social Medicine, Dresden University of Technology, Dresden, Germany; ^4^Department of Child Health and Development, Norwegian Institute of Public Health, Oslo, Norway; ^5^Department Woman-Mother-Child, Faculty of Biology and Medicine, Lausanne University Hospital, Lausanne, Switzerland

**Keywords:** birth-related posttraumatic stress disorder, parent-infant bonding, perinatal mental health, postpartum, longitudinal, cohort, fathers, childbirth

## Abstract

The parent-infant bond following childbirth is an important facilitator of optimal infant development. So far, research has mainly focused on mother-infant bonding. Data on fathers are still sparse. Parental mental health, such as posttraumatic stress symptoms (PTSD), may influence mother-infant relations and/or interactions. There is evidence that both parents can experience PTSD symptoms following childbirth (PTSD-CB). The aim of this study is to investigate the prospective relationship between parental PTSD-CB symptoms at 1 month postpartum and perceived parent-infant bonding at 3 months postpartum, while adjusting for antenatal confounders. A subsample was used for this study (*n*_Totalsample_ 488, *n*_mothers_ = 356, *n*_fathers_ = 132) of an ongoing prospective cohort study. Future parents awaiting their third trimester antenatal appointments at a Swiss university hospital were recruited. Self-report questionnaires assessed PTSD-CB symptoms and psychological distress at 1 month postpartum, and parent-infant bonding at 3 months postpartum. Confounders included antenatal PTSD symptoms and social support measured *via* self-report questionnaires, and gestity and gestational age, extracted from medical records. Using structural equation modeling, the predictive ability of PTSD-CB symptoms at 1 month postpartum on parent-infant bonding at 3 months postpartum was assessed for both parents respectively. Maternal PTSD-CB symptoms at 1 month postpartum were found to be negatively prospectively associated with mother-infant bonding at 3 months postpartum; however, this effect disappeared after adjusting for psychological distress at 1 month postpartum. No such effects were found for fathers. There was no evidence of mediation of the relationship between parental PTSD-CB symptoms at 1 month postpartum and parental-infant bonding at 3 months postpartum *via* psychological distress at 1 month postpartum. However, such a mediation was found for maternal intrusion and hyperarousal symptom subscales. Results expand the current literature on the impact of PTSD-CB on parent-child relations to also include fathers, and to a community sample. Any adverse effects of mental health symptoms on parent-infant bonding were evidenced by 3 months postpartum only for mothers, not fathers. Our results may inform the development of prevention/intervention strategies.

## Introduction

Bonding has been described as the mother’s feelings towards her child; a process that is differentiated from observable attachment behaviors (1). The mother-infant bond following childbirth is an important facilitator of optimal infant development (2). It is thought to first appear during pregnancy or shortly after birth and then to develop over the first few months postpartum (3, 4). Thus, it is important to understand the factors that may shape it, both during the prenatal and postpartum period. So far, research has mainly focused on the bonding between the mother and her newborn (e.g., Farré-Sender, 2018). Although the role of the father has recently received more attention, data on this issue are still sparse and more research is needed (5). This study therefore specifically investigates the bonding with the infant by both parents.

Difficulties in establishing the mother-infant bond may result in significant negative alterations in the infant’s developing brain and increase the risk for developmental, emotional, and behavioral difficulties, as well as long-term impairment of the mother-child relationship, which in extreme cases can result in complete rejection of the infant (6, 7). A variety of studies has shown that maternal mental health, and postpartum depression in particular, may interfere with bonding (8). This may partly be explained by disrupted maternal sleep (9). Some researchers propose that bonding difficulties develop secondary to an untreated primary mother’s postpartum depression, whereas others suggest that these can also occur in women who have not suffered from maternal depression (7). Regarding fathers, one small study (n = 66) showed that affective symptoms at 3 months postpartum was not associated with father-infant bonding at 3 months postpartum but predicted father-infant bonding at 15 months postpartum (10). However, more evidence in a larger sample is needed.

Another mental health issue that may interfere with bonding is birth-related posttraumatic stress disorder (PTSD-CB) that women and their partners may develop if a direct or indirect exposure to the threatened death or severe injury of the mother and/or her baby took place (11, 12). PTSD-CB affects between 3% and 4% of women after birth at diagnostic levels in community samples, and around 16% to 19% of women in high-risk groups, e.g., after preterm birth or neonatal death (13, 14). In addition, a considerable percentage of women experiences clinically significant PTSD-CB symptoms, even though they do not reach the diagnostic threshold level (15). Evidence shows that subthreshold symptomatology may also significantly impact on women’s functioning, particularly if they are having symptoms of re-experiencing (16). Furthermore, fathers may also be traumatized by the birth they witnessed and develop PTSD-CB, although prevalences are likely to be lower [e.g., (17)].

Some studies have examined the impact of maternal PTSD-CB on mother-infant bonding, based on the assumption that PTSD-CB symptoms may limit maternal sensitivity (18), but the findings are mixed [see (19), for a review]. Results seem to vary depending on the instrument used (20), how postpartum PTSD is defined (21), or whether comorbid symptoms of depression are considered (22).There is also evidence that maternal PTSD-CB symptoms may affect mother-infant interactions (23) and mother-infant bonding in infants born at risk, such as preterm infants (24) or infants with perinatal asphyxia (25). One recent cross-sectional web-based survey reported a medium indirect relationship between (what the authors call) “postnatal maternal PTSD symptoms” and the mother-infant bond that was fully mediated by depression symptoms (26). The authors argued that the impact of postnatal PTSD symptoms on maternal affect, cognition and behavior, and, in turn, on bonding might be less important than that of depression (26). Regarding fathers, the evidence is still limited. Two studies found no impact of fathers’ PTSD-CB symptoms on father-infant bonding (25). However, these studies are limited by their small sample size (10) and their retrospective design (25). It is thus important that, as a next step, the prospective relationship between PTSD-CB symptoms and parent-infant bonding be examined in a larger population-based cohort of both mothers and fathers.

PTSD symptoms form several clusters; namely, intrusions or re-experiencing (e.g., intrusive thoughts/images), avoidance (e.g., non-attendance at hospital appointments if the birth took place at the hospital), hyperarousal (e.g., hypervigilance with regards to her baby) and negative cognitions and mood (e.g., ‘It’s my fault that obstetric complications occurred’) (11). There may be reason to suspect these clusters having differential relationships with parent-infant bonding. Mothers with PTSD have shown fearful (withdrawing or avoidance) behavior when interacting with their infant (27). These behaviors are more likely to be related to the avoidance symptom cluster, where parents avoid reminders of the traumatic event, which in this case of PTSD-CB could be their baby, than intrusive or hyperarousal symptoms. Indeed, PTSD symptom clusters have been found to have differential influences on other maternal behaviors that may facilitate bonding, such as breastfeeding (23). While both intrusion and avoidance symptoms were found to be associated with noninitiation of breastfeeding, only avoidance symptoms were associated with not continuing to breastfeed at 1 year postpartum. There has been little attention given to potential differential impact of parental PTSD-CB symptom clusters on parent-infant bonding, which may be informative for intervention development.

Several factors may have an influencing role on the prospective relationship between PTSD-CB symptoms and parent-infant bonding. Obstetric factors, such as mode of childbirth, may cause a prolonged separation from the infant and thus affect the formation of the parent-infant bond in the first hours after childbirth. Research comparing vaginal birth and either elective or emergency caesarean sections reported a delay in the creation of the mother-infant contact (28). A meta-analysis concluded that following a caesarean section, mothers experienced a longer delay until their first interaction with their infants, had less positive reactions to them after birth, and interacted with them less at home compared to vaginal births (29). However, this may be different for fathers who often stay with the newborn whilst the mother has to undergo medical procedures following obstetric complications during childbirth.

To identify the effects specific to PTSD-CB, it is essential to adjust for past stress and trauma exposure, as well as past PTSD (including pre-partum PTSD) of both parents (5). This prospective study allows us to adjust for these variables, such as lifetime trauma exposure, previous traumatic birth experience, antenatal PTSD symptoms, and antenatal psychological distress previously shown to be important risk factors for PTSD-CB, as well as social support, an important protective factor (30).

In a prospective population-based cohort of mothers and fathers, we examined three main research questions: (1) Are parental PTSD-CB symptoms (assessed at 1 month postpartum) prospectively associated with parent-infant bonding (assessed at 3 months postpartum)? We expected that parental PTSD-CB symptoms would show a negative prospective association with parent-infant bonding. (2) Does psychological distress (assessed at 1 month postpartum) mediate the relationship between PTSD-CB symptoms (assessed at 1 month postpartum) and bonding (assessed at 3 months postpartum)? The relationships amongst parental PTSD-CB symptoms at 1 month postpartum, concurrent psychological distress, and parent-infant bonding at 3 months postpartum is not clear: concurrent psychological distress could have a confounding effect or act as an intermediary variable. Therefore, we would first check whether concurrent psychological distress is exerting any effect on the association between PTSD-CB symptoms at 1 month postpartum and parent-infant bonding at 3 months postpartum through checking its role as a confounding factor. If we found evidence it was implicated, we would then test it as a mediator. We expected that psychological distress would mediate the relationship between PTSD-CB symptoms and bonding. Each of these research questions was examined separately for mothers and fathers. We adjusted for important antenatal confounders in all analyses (see below).

## Materials And Methods

### Design

Participants in this study represent a sample of participants from the Lausanne Perinatal Wellbeing Cohort, an ongoing prospective population-based cohort study, which commenced data collection in 2013. Within this cohort, women and their partners are recruited during their third trimester of pregnancy while they wait for their antenatal appointment at a Swiss University Hospital. The study is presented to them by a member of the research team and they are able to ask questions before they give their signed consent. Women and their partners who agree to participate are asked to answer questionnaires during the third trimester (T1/baseline) of their pregnancy, 1 week postpartum (T2), 1 month postpartum (T3), 3 months postpartum (T4), and 6 months postpartum (T5). Medical data concerning the childbirth and the baby are extracted postpartum. The measures used to answer the research questions in this study are taken from time points T1 – T4. Inclusion criteria for this cohort were: future parents in their third trimester, sufficient French language skills to understand the information sheets and questionnaires, and being aged 18 years or older. Although both mothers and partners were approached to take part, participation in the study was not dependent on both future parents agreeing to take part.

### Participants

Data from the Lausanne Perinatal Wellbeing Cohort were downloaded in June 2019 and contained 675 participants who had answered the questionnaires at, at least, one of the five time points. For the present study, we excluded mothers and partners of twins or multiple births (n = 21), as bonding and its associations may be different for multiple versus unique births. There was an extra inclusion criterion for partners, that they should have been present at the birth. This was determined by mothers’ response to the question “Who was present at the birth?” or the partners’ response to the question “Were you present at the birth?” Where a conflict in responses occurred between a couple on these questions, partners’ answers would have been prioritized; however, no such conflict occurred within this sample. As this question is a recent addition to the cohort, it was not available for all participants. Where this question was not available, the partner’s presence at the birth was inferred *via* completion of the questionnaire “Peritraumatic Dissociation Experiences Questionnaire” [French Version; PDQ-FR (31)], answered at 1 week postpartum. The rationale here was that given the content of the questions and the instruction posed, the partner could not have answered this questionnaire unless they were present at the birth.

Multiparous and primiparous parents were included in this study. To ensure that there were no differences within this sample in bonding, average bonding scores were compared between multiparous and primiparous groups. As no difference was found between multiparous and primiparous parents in scores from measures of bonding taken at 3 months postpartum (*t*(364) = −0.49, *p* = 0.6281), it was deemed appropriate to include both sets of parents.

**Figure 1:**
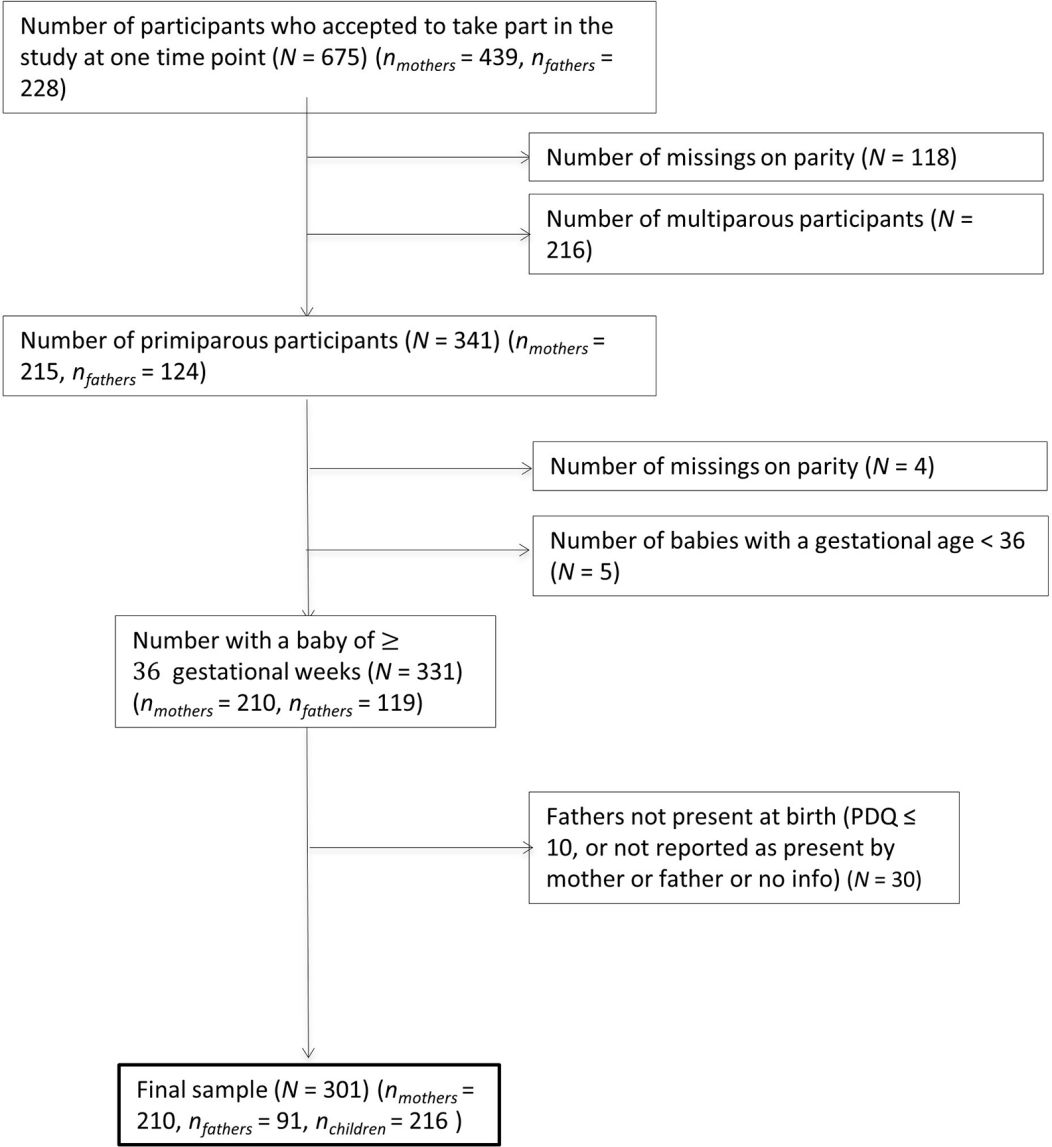
Flow of study participants.

After applying inclusion/exclusion criteria to the cohort, 488 participants were included in the present study (*n*_mothers_ = 356, *n*_fathers_ = 132, *n*_childbirths_ = 368). Participants were on average 32.94 years old (*SD* = 4.49, *min* = 18, *max* = 49). There was a significant difference in age between mothers and fathers in the sample (*t*(290) = −3.20, *p* = .001, *d* = -.30), where fathers (*M* = 34.04, *SD* = 4.5) were slightly older than mothers (*M* = 32.55, *SD* = 4.4). Although female partners were eligible to take part in the cohort, there were no female partners within this sample after the inclusion criteria were applied (see **Figure 1** for study flowchart). Demographics of the sample can be found in **Table 1**. This table shows that the majority of participants in the sample were Swiss, university educated with managerial level jobs, and living as a cohabiting or married couple. Parity (number of live births) ranged between 0 (59%) and 3 (2.5%), with the majority of the sample being first-time parents and an average gestity (number of pregnancies) of 1.86 (*SD* = 1.06, *min* = 0, *max* = 8). The childbirths in the sample were on average full term pregnancies (*Mgestationalage* = 278.93 days/39.84 weeks, *SD* = 16.01 days), 56% represented spontaneous vaginal deliveries, 16% represented planned instrumental deliveries, and 26% represented unplanned instrumental deliveries. The babies from these births were majority male (54%).

**Table 1:** Demographic data of the sample by gender of parents.

		All (*n* = 488)	Mothers (*n* = 356)	Fathers (*n* = 132)
Nationality (99%)		n (%)	n (%)	n (%)
	Swiss	276 (57)	198 (56)	78 (60)
	European	176 (36)	127 (36)	48 (37)
	Non-European	34 (7)	29 (8)	5 (4)
Educational level (98%)				
	Primary school	2 (.4)	2 (.6)	–
	Middle school	20 (4)	16 (5)	4 (3)
	Secondary school	25 (5)	21 (6)	3 (3)
	Apprenticeship	96 (20)	74 (21)	22 (17)
	University	308 (63)	218 (63)	90 (71)
	Other	25 (5)	18 (5)	7 (6)
	Unskilled labor	13 (3)	11(3)	2 (2)
Marital status (99%)				
	Single	120 (25)	83 (23)	37 (28)
	Couple/married	354 (73)	262 (74)	92 (70)
	Divorced	2 (.4)	2 (1)	–
	Separated	7 (1.4)	5 (1)	2 (2)
	Widow/widower	2 (.4)	2 (.6)	–

### Measures

#### Variables of Interest

##### Mother-to-Infant Bonding Scale (MIBS)

This 8-item questionnaire assesses the parents’ feelings towards the new baby in the first few weeks after birth (1). Eight adjectives are rated on a scale from 0 = *very much* to 5 = *not at all*. Scores were summed to create a total score, with a higher score indicating worse mother-to-infant bonding. The MIBS has shown good initial psychometrics (1). The validated French version was used in this study (32). This measure has also been previously used with fathers to asses bonding, showing adequate internal reliability across both mothers and fathers (25). Internal reliability within this sample for the total score was adequate when measured at T4/3 months postpartum (α_Total_ = .73; α_Mothers_ = .73; α_Fathers_ = .73).

##### Post Traumatic Diagnostic Scale: French Version (PDS-F)

The 17 items of the PDS-F were used to assess self-reported severity of PTSD symptoms (according to DSM-IV) related to childbirth (PTSD-CB) (33). Participants were asked to respond to the items with particular reference to their childbirth or caesarean. Participants respond to the items by indicating if they experience the symptom on a scale of 0 = *not at all or one time only* to 3 = *5 times a week or more*. Item scores can be summed up to create a total score (0 to 51), here latent scores will be created following the results of the CFA. The original English PDS has shown good psychometric properties including specificity and sensitivity (33) and there is preliminary validation evidence for the French version (34). The PDS-F was administered at T1/third trimester and T3/1 month postpartum. PDS-F assessment from T1 was used as a covariate in the analysis. Internal reliability within this sample was excellent across time points (α_Total_ = .88 -.90; α_Mothers_ = .88 -.92; α_Fathers_ = .86 -.88).

#### Potential Confounders

##### Hospitalized Anxiety and Depression Scale: French Version (HADS-F)

This self-report questionnaire measures severity of anxiety and depression symptoms during the last week (35). The HADS contains an anxiety subscale and a depression subscale consisting of 7 items each, scored on a 4-point scale. The total score is calculated as a sum of all items of both subscales to assess psychological distress (0 to 52). A higher score indicates higher distress (35). Good psychometric characteristics have been reported for the French version (36). Internal reliability within this sample for the total score was adequate when assessed at T1/third trimester (antenatal) (α_Total_ = .78; α_Mothers_ = .78; α_Fathers_ = .80 and at T3/1 month postpartum (α_Total_ = .86; α_Mothers_ = .85; α_Fathers_ = .85).

##### Medical Outcome Study Social Support Scale (MOS)

The MOS is a brief 20-item measure of perceived social support (37). One question asks respondents to estimate how many close friends and relatives they have. In the 19 remaining items, respondents are asked to indicate how often different types of support are available to them, using a 5-point Likert scale of 1 = *none of the time* to 5 = *all of the time*. The 19 Items load onto four factors representing different types of support: Emotional/informational (8 items), tangible (4 items), affectionate (3 items), and positive social interaction (3 items). Scales were combined to create a total score of social support by averaging across all items. Higher scores indicate the more frequent availability of different types of support if needed. The measure has shown evidence of internal reliability, stability over time, and construct validity (37). There is currently no validation French translation of this measure; thus, a French translation was created following Wild, Grove (38)’s principles of good practice for translation and cultural adaption. This measure was assessed at T1/third trimester. Internal reliability within this sample for the total score was excellent (α_Total_ = .95; α_Mothers_ = .96; α_Fathers_ = .94).

##### Obstetric History

The number of children from the current pregnancy, the number of previous pregnancies (gestity), and the number of living offspring (parity) were extracted from medical records postpartum. Gestity was extracted as a potential confounder.

##### Birth-Related Information

The mode of delivery was extracted from medical records postpartum and categorized as one of the following: spontaneous delivery (0; vaginal delivery), planned instrumental delivery (1; i.e., planned caesarian section), and unplanned instrumental delivery (2; e.g., emergency caesarian section, forceps or ventouse delivery). Gender of the child was also extracted. Gestational age (days) of the child was extracted for investigation as a potential confounder

#### Descriptive Variables

##### Psychological History

Five single yes/no items were asked to assess the history of psychological difficulties, current psychological difficulties, previous trauma, previous experience of a traumatic birth, and previous loss of a child through: miscarriage, still birth, or as a newborn. The items regarding history of psychological difficulties, current psychological difficulties, and previous trauma included a follow up question if a “yes” response was given, requesting a description of the difficulty/trauma. All these items were asked at T1/third trimester

##### Demographics

Sociodemographic data were also collected from participants at T1/third trimester. Demographic data used in this study included: marital status [single (1); married or co-habiting (2); separated (3); divorced (4); widow/widower (5); other (6)], age, nationality [Swiss (1); European (2); Non-European (3)], profession, and educational level [Primary school (1); Middle school (2); Secondary school (3); Apprenticeship (4); University (5); Other (6)] Finally, whether someone was present at the birth alongside the mother and who this person was, was asked at T2 (1 week postpartum).

### Statistical Approach

To identify which of the potential confounders should be included in the models, bivariate correlations between these potential confounders (antenatal PTSD symptoms, antenatal social support, gestity, gestational age, and general psychological distress at 1 month postpartum) and the variables of interest (PTSD-CB symptoms at 1 month postpartum and parent-infant bonding at 3 months postpartum) were conducted. When normality was assessed, parent-infant bonding scores and PTSD-CB symptoms score at 1 month postpartum were found to be skewed. Amongst the potential confounders, scores of antenatal PTSD symptoms, antenatal social support, and gestational age were all found to be skewed. Therefore, non-parametric correlations were used to assess associations.

To achieve the simplest model and to maximise the power in the sample, only the primary variables of interest, i.e., parent-infant bonding at 3 months postpartum and PTSD symptoms at 1 month postpartum, were modeled as latent variables. All other variables included in the model were modeled as observed variables using the total scores of the questionnaires. To check whether latent variables were appropriate for these variables, measurement models were created to check the model fit of mother/father-infant bonding at 3 months postpartum and PTSD symptoms at 1 month postpartum for mothers and fathers, respectively. To obtain a good fit to the data, items 6, 7, and 8 were removed from the PDS-F at 1 month postpartum for mothers, and item 2 was removed for the PDS-F at 1 month postpartum for fathers. Theoretically, it makes sense that these items did not contribute to the latent factor in the context of PTSD-CB. Items 6, 7, and 8 refer to avoidance behaviors of reminders related to the trauma. For mothers, it is difficult to avoid reminders of their birth or their babies. Item 2 refers to bad dreams or nightmares. Fathers were generally reporting that this was not a symptom they experienced, regardless of whether other symptoms were present or not. In this sample, it is therefore not a good indicator of PTSD-CB and should be removed for fathers. Following these omissions, a model, where items of the parent-infant bonding scale loaded onto one factor (Mothers: coefficients ranged from .33–.92; Fathers: coefficients ranged from: .58–.82), showed a good fit to the data (Mothers: CFI = .97; TLI = .96; RMSEA = .07; Fathers: CFI = .99; TLI = .99; RMSEA = .03). A model, where items of the PDS-F were loaded onto three factors, namely intrusions (Mothers: coefficients ranged from: .77–.86; Fathers: coefficients ranged from: .75–1.02), Hyperarousal (Mothers: coefficients ranged from .62–.86; Fathers: coefficients ranged from: .69–.79), avoidance (Mothers: coefficients ranged from: .70–.83; Fathers: coefficients ranged from: .61–.93) was assessed. These models showed a good fit to the data for both mothers (CFI = .97; TLI = .96; RMSEA = .06) and fathers (CFI = .98; TLI = .97; RMSEA = .05).

The structural equation modeling approach allowed us to identify pertinent confounders for each of the variables of interest. Confounders were added simultaneously to the model for each of the research questions to estimate the unique contributions of predictor variables on the latent factor parent-infant bonding. Regarding the question about mediation, our variable of interest (parental PTSD-CB symptoms) and our proposed mediator (parent general psychological distress) were measured concurrently (1 month postpartum). Given this, to reduce potential bias and to accurately evaluate the association between parent PTSD-CB symptoms and parental general psychological distress, it was necessary to adjust for prior (antenatal) parental PTSD symptoms and parental general psychological distress (39). Bootstrapping with 1000 iterations was used to test for the significance of the indirect effects within the mediation models.

Robust maximum likelihood estimation was used to account for the non-normality amongst the variables. Percentage missing for items at baseline, 1 month postpartum, 3 months postpartum, and through the demographics items ranged from .1% to 1.7% for mothers, and .1% and 3.2% for fathers. Missingness was accounted for using full information maximum likelihood.

All analyses were conducted using R studio version 1.2.5033 (40) and R version 3.6.2 (41) with the Lavaan package (42). Packages Visdat (43) and psych (44) were used to help prepare the data and obtain descriptive and psychometric properties of the questionnaires.

## Results

**Table 2** presents the descriptive statistics of the variables included in this study of the sample, as well as mothers and fathers separately. Gender differences in perinatal mental health variables, including PTSD-CB and psychological distress from the Lausanne Perinatal Wellbeing Cohort have been investigated and discussed elsewhere (17). In general, these results indicate that mothers have higher symptom scores on mental health variables and more probable diagnoses within this cohort at the antenatal and 1 month postpartum time point.

**Table 2:** Descriptive statistics of all primary variables of interest and confounder variables for the complete sample and by parent gender.

		All (*M, SD*)	Mothers (*M, SD*)	Fathers (*M, SD*)
*Primary Variables of Interest*				
	Bonding at 3 months	1.72 (2.25)	1.67 (2.26)	1.83 (2.23)
	PTSD-CB symptoms at 1 month^1^	5.80 (6.49)	6.66 (6.90)	3.77 (4.87)
*Covariates*				
	Antenatal social support	8.55 (1.36)	8.59 (1.38)	8.46 (1.32)
	Psychological distress at 1 month	9.96 (6.11)	11.32 (6.12)	8.05 (5.34)
	Antenatal PTSD symptoms	6.50 (7.24)	6.99 (7.61)	5.12 (5.90)
	Antenatal psychological distress^2^	10.30 (5.32)	11.14 (5.28)	8.2 (4.89)

^1^Total scores were created based on the items included in the latent variables following the CFA. ^2^Only used as a covariate for mediation analyses.

### Investigation of Confounders

All potential confounders showing a significant correlation with the variables of interest were included in the models (see **Table 3**, correlations in bold). As expected, maternal psychological distress at 1 month postpartum was associated with maternal PTSD-CB symptoms at 1 month postpartum and with mother-infant bonding at 3 months postpartum (see **Table 3**). It is interesting to note that there was no association found between paternal psychological distress at 1 month postpartum and father-infant bonding at 3 months postpartum.

**Table 3:** Bivarate pearson correlations between variables of interests and potential covariates for mothers and fathers.

	PTSD-CB symptoms at 1 month	Antenatal PTSD symptoms	Psychological Distress at 1 month	Antenatal Social Support	Gestational age	Gestity
*Mothers*						
Mother-infant bonding at 3 months	.21^**^	.10	.35^***^	−.18^**^	−.03	−.03
PTSD-CB symptoms at 1 month	–	.50^***^	.72^**^	−.25^***^	−.03	.07
Antenatal PTSD symptoms	–	–	.51^**^	−.32^***^	−.001	.20^***^
Psychological distress at 1 month	–	–	–	−.30^***^	−.08	.13
Antenatal social support	–	–	–	–	.006	−.15^**^
Gestational age	–	–	–	–	–	−.10
*Fathers*						
Bond	.27^*^	.18	.25	−.32^**^	−.01	−.02
PTSD-CB 1m	–	.43^***^	.40^***^	−.06	.12	−.20
Antenatal PTSD symptoms	–	–	.50^***^	−.32^**^	-.03	−.05
Psychological distress at 1 month	–	–	–	−.32^***^	.07	.02
Antenatal social support	–	–	–	–	.02	−.07
Gestational age	–	–	–	–	–	−.12

PTSD-CB, childbirth-related PTSD symptoms; PTSD, posttraumatic stress disorder. ^*^p < .05; ^**^p < .01; ^***^p < .001.

### Prospective Association Between Parental Birth-Related PTSD Symptoms and Parent-Infant Bonding

**Figure 2:**
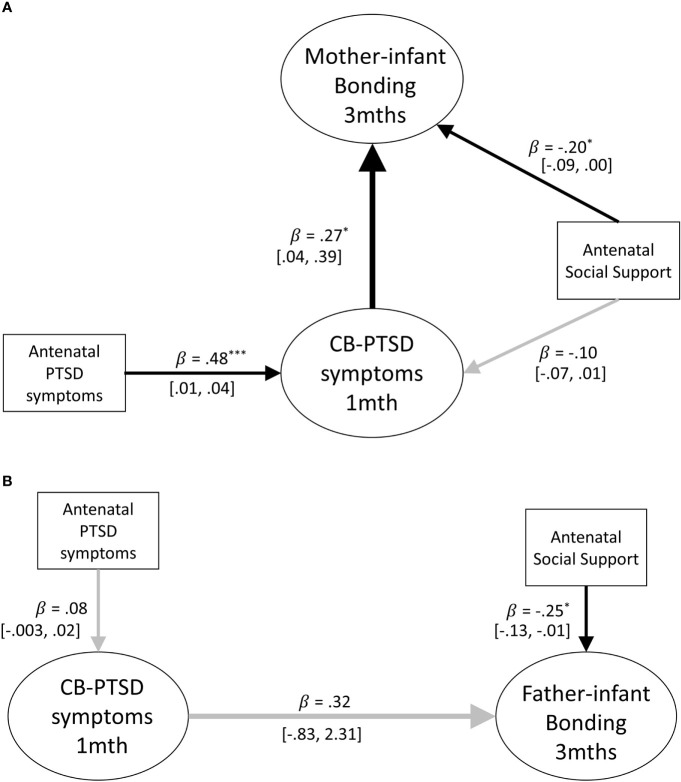
**(A)** Path model of the prospective prediction of mother-infant bonding at 3 months by postpartum PTSD symptoms at 1 month. Antenatal social support and PTSD symptoms are covariates. Black lines indicate significant pathways, grey lines indicate non-significant pathways. Standardized coefficients are reported and 95% confidence intervals. p < .05; ^**^p < .01; ^***^p < .001. **(B)** Path model of the prospective prediction of father-infant bonding at 3 months by postpartum PTSD symptoms at 1 month. Antenatal social support and PTSD symptoms are included as covariates. Black lines indicate significant pathways, grey lines indicate non-significant pathways. Standardized coefficients and 95% confidence intervals are reported. PTSD-CB, childbirth-related posttraumatic stress. ^*^p < .05; ^**^p < .01; ^***^p < .001.

Structural equation models were constructed to assess whether parental birth-related PTSD symptoms at 1 month postpartum, were prospectively associated with parent-infant bonding at 3 months postpartum for mothers and fathers, respectively (see **Figures 2A, B**). The pathway of interest to the first research question is the path between parental PTSD-CB symptoms at 1 month postpartum and parent-infant bonding at 3 months postpartum. To complete answering this question, respective cross associations between mother/father PTSD-CB symptoms and father/mother-infant bonding were investigated. Participants were included in this investigation if data from both members of a couple were available.

#### Mothers

The model for mothers accounted for 14% of the variance of the mother-infant bonding scores at 3 months postpartum, indicating that maternal PTSD-CB symptoms at 1 month postpartum were prospectively associated with mother-infant bonding. The co-efficient indicates that higher maternal birth-related PTSD symptoms at 1 month postpartum were prospectively associated with worse mother-infant bonding at 3 months postpartum (see **Figure 2A**).

#### Fathers

The model for fathers accounted for 24% of the variance of the father-infant bonding scores at 3 months postpartum, indicating that paternal PTSD-CB symptoms at 1 month postpartum were not prospectively associated with father-infant bonding at 3 months postpartum (see **Figure 2B**). The only significant pathway in the model for fathers was the pathway between the covariate antenatal social support and father-infant bonding at 3 months postpartum.

#### Cross-Associations

Data were available for both members of a couple for 77 couples. Given this small sample size, the cross-associations between parental PTSD-CB symptoms at 1 month postpartum and parent-infant bonding at 3 months postpartum were investigated using Spearman correlations. No correlation was found between maternal PTSD-CB symptoms at 1 month postpartum and father-infant bonding at 3 months postpartum (r = -.03, p = .789). However, there was a significant positive association between paternal PTSD-CB symptoms at 1 month postpartum and mother-infant bonding at 3 months postpartum (r = .27, p = .01) with a small effect. This association suggests that higher paternal PTSD-CB symptom scores at 1 month postpartum are related to worse mother-infant bonding at 3 months postpartum.

### Mediation of Parental Birth-Related PTSD Symptoms and Parent-Infant Bonding *via* Parent General Psychological Distress

#### Adjusting for General Psychological Distress at 1 Month Postpartum

**Figure 3:**
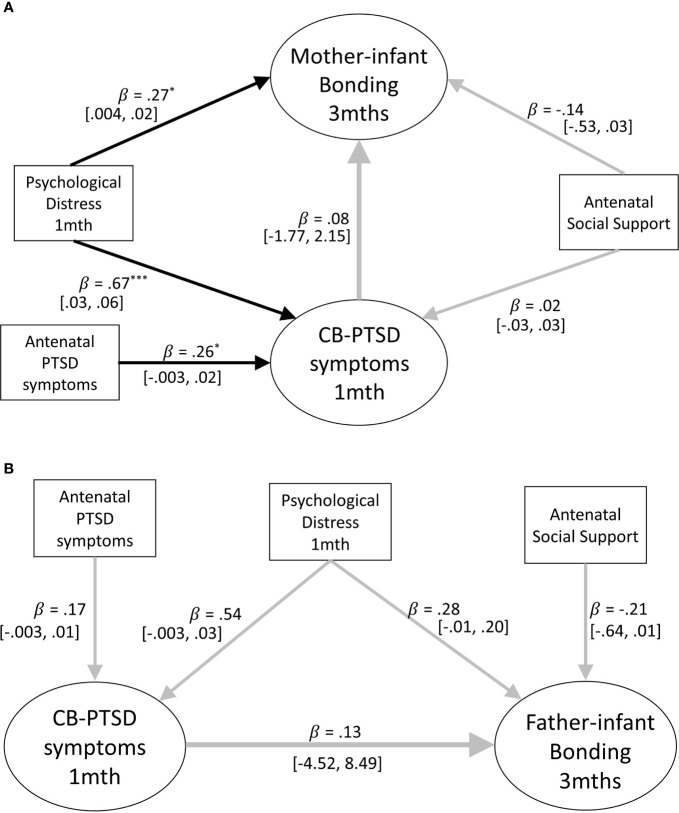
**(A)** Path model of the prospective prediction of mother-infant bonding at 3 months by postpartum symptoms PTSD at 1 month, adjusting for concurrent psychological distress. Antenatal social support and PTSD symptoms are also included as covariates. Black lines indicate significant pathways, grey lines indicate non-significant pathways. Standardized coefficients and 95% confidence intervals are reported. ^*^p < .05; ^**^p < .01; ^***^p < .001. **(B)** Path model of the prospective prediction of father-infant bonding at three months by postpartum PTSD symptoms at one month adjusting for concurrent psychological distress. Antenatal social support and PTSD symptoms are also included as covariates. Black lines indicate significant pathways, grey lines indicate non-significant pathways. Standardized coefficients and 95% confidence intervals are reported. PTSD-CB, childbirth-related posttraumatic stress. ^*^p < .05; ^**^p < .01; ^***^p < .001.

As a first step to assess whether concurrent psychological distress at 1 month postpartum is implicated in the association between parental PTSD-CB symptoms and parent-infant bonding at 3 months postpartum, we first assessed concurrent psychological distress as potential confounder. Concurrent psychological distress may act as a confounder in the sense that presence of such distress may increase the risk of worse parent-infant bonding for those with PTSD-CB symptoms. Thus, structural equation models were constructed to assess whether parental PTSD-CB symptoms at 1 month postpartum were prospectively associated with parent-infant bonding at 3 months postpartum after adjusting for general psychological distress at 1 month postpartum for mothers and fathers, respectively (see **Figures 3A, B**). The pathway of interest to the second research question is the path between parental PTSD-CB symptoms at 1 month postpartum and parent-infant bonding at 3 months postpartum.

##### Mothers

The model for mothers accounted for 15% of the variance of mother-infant bonding scores at 3 months postpartum, indicating that maternal PTSD-CB symptoms at 1 month postpartum were no longer predictive of mother-infant bonding once maternal general psychological distress at 1 month postpartum was added to the model (see **Figure 3A**). The model also suggests that maternal general psychological distress was associated with mother-infant bonding, where higher general psychological distress at 1 month postpartum was predictive of worse mother-infant bonding at 3 months postpartum (see **Figure 3A**). Thus, it appears maternal psychological distress may be implicated in the association between maternal PTSD-CB symptoms at 1 month postpartum and parent-infant bonding at 3 months postpartum.

##### Fathers

The model for fathers accounted for 24% of the variance of father-infant bonding scores at 3 months postpartum; however, neither paternal PTSD-CB symptoms, nor paternal general psychological distress at 1 month postpartum were predictive of father-infant bonding at 3 months postpartum (see **Figure 3B**). Given the lack of impact testing paternal concurrent psychological distress as a confounder has had on the model, we were not justified to test concurrent psychological distress as a mediator for fathers.

#### Assessing Mediation of Parental Birth-Related PTSD Symptoms and Parent-Infant Bonding *via* Parent General Psychological Distress

**Figure 4:**
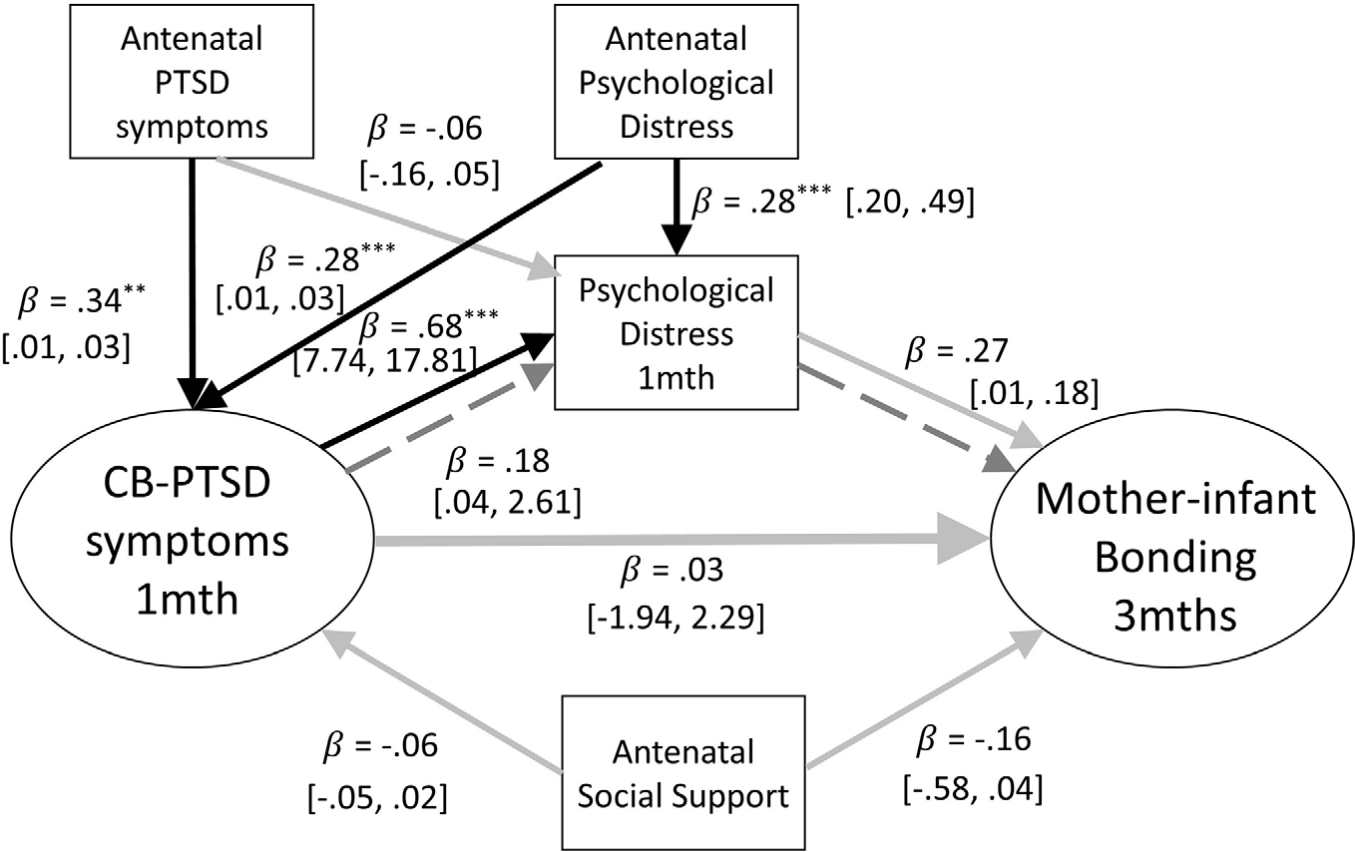
Path model of the mediation of the relationship between mother-infant bonding at three months and postpartum PTSD symptoms at one month by concurrent psychological distress. Antenatal social support and PTSD symptoms are also included as covariates. Black lines indicate significant pathways, grey lines indicate non-significant pathways, dashed lines signify the indirect effect. Standardized coefficients and 95% confidence intervals are reported. PTSD-CB, childbirth-related posttraumatic stress disorder. ^*^p < .05; ^**^p < .01; ^***^p < .001.

As the assessments of concurrent psychological distress are suggestive that this variable is implicated in the association between parental PTSD-CB symptoms at 1 month postpartum and parent-infant bonding at 3 months postpartum, it was then tested as a mediator to further clarity the nature of this role. Structural equation models were constructed to assess whether parental psychological distress at 1 month postpartum mediated the relationship between parental PTSD-CB symptoms at 1 month postpartum and parent-infant bonding at 3 months postpartum, for mothers and fathers respectively (see **Figure 4**). The pathway of interest to the third research question is the indirect pathway between parental PTSD-CB symptoms at 1 month postpartum parent-infant bonding *via* parent general psychological distress.

##### Mothers

The model for mothers accounted for 13% of the variance of mother-infant bonding scores at 3 months postpartum. The path from PTSD-CB symptoms at 1 month postpartum to psychological distress at 1 month postpartum was significant, and the path from psychological distress to mother-infant bonding at 3 months postpartum was close to significance (*p* = .057; see **Figure 4**). However, no significant indirect pathway was found, suggesting that there was no mediation by maternal general psychological distress (see **Figure 4**). The total direct effect was also not significant (*B* = 1.20, *β* = .21, *p* = .075, 95% CI = [.04, 2.61]). The direct path between maternal PTSD-CB symptoms at 1 month postpartum and maternal psychological distress at 1 month postpartum was significant. The coefficient indicates that higher PTSD-CB symptoms at 1 month postpartum was associated with higher psychological distress at 1 month postpartum. There were also significant direct paths concerning the confounders. Both, antenatal PTSD symptoms and antenatal psychological distress were prospectively associated with maternal PTSD-CB symptoms at 1 month postpartum, whereas higher antenatal symptoms were prospectively associated with higher PTSD-CB scores at 1 month postpartum. Finally, antenatal maternal psychological distress was prospectively associated with maternal psychological distress at 1 month postpartum, where higher antenatal symptoms of psychological distress was prospectively associated with higher psychological distress at 1 month postpartum (see **Figure 4**).

### Subscales of the PDS-F: Intrusion, Hyperarousal, and Avoidance

All models above were also run for each of the subscales of the PDS-F reflecting PTSD-CB symptom clusters of intrusions, avoidance, and hyperarousal, for mothers and fathers, respectively. For brevity, only patterns and significant findings directly relevant to the research questions are reported. Figures related to all models tested can be found in Supplementary materials.

#### Mothers

Models investigating whether symptom subscales at 1 month postpartum were prospectively associated with mother-infant bonding at 3 months postpartum explained 8% to 15% of the variance on mother-infant bonding at 3 months postpartum. The only subscale to show an association between maternal PTSD-CB symptoms and mother-infant bonding was the avoidance subscale (*B* = .18, *β* = .30, *p* = .018, 95% CI = [.04,.34]). As with the full scale of PTSD-CB symptoms, when maternal general psychological distress was added to the model, this association disappeared.

**Figure 5:**
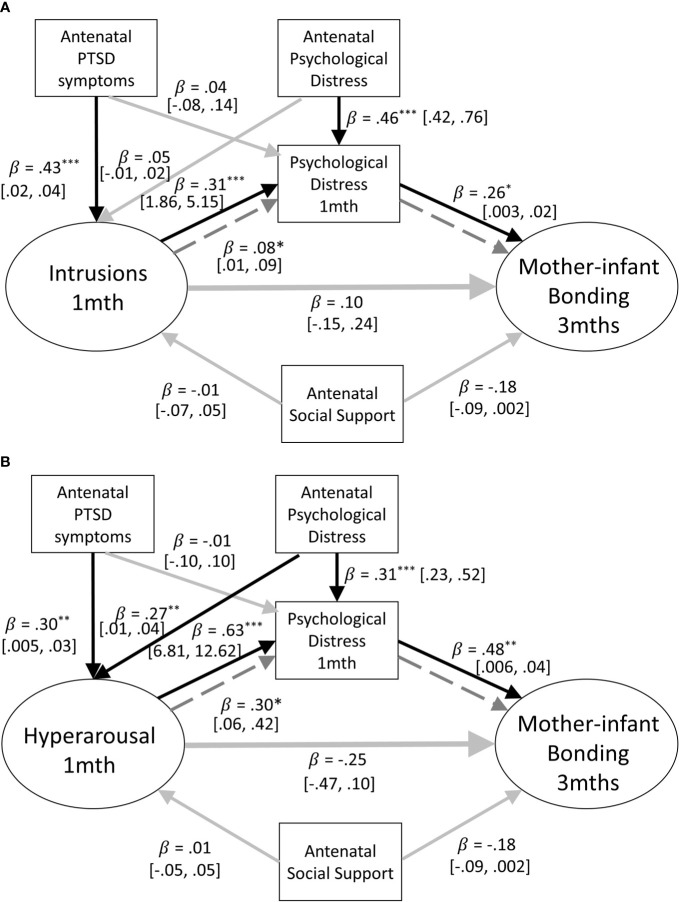
**(A)** Path model of the mediation of the relationship between mother-infant bonding at 3 months and postpartum intrusion symptoms at one month by concurrent psychological distress. Antenatal social support and PTSD symptoms are also included as covariates. Black lines indicate significant pathways, grey lines indicate non-significant pathways, dashed lines signfiy the indirect effect. Standardized coefficients and 95% confidence intervals are reported. ppPTSD, Postpartum Post Traumatic Stress Disorder. ^*^p < .05; ^**^p < .01; ^***^p < .001. **(B)** Path model of the mediation of the relationship between mother-infant bonding at three months and postpartum hyperarousal symptoms at one month by concurrent psychological distress. Antenatal social support and PTSD symptoms are also included as covariates. Black lines indicate significant pathways, grey lines indicate non-significant pathways, dashed lines signfiy the indirect effect. Standardized coefficients and 95% confidence intervals are reported. PTSD-CB, Childbirth-related Posttraumatic Stress. ^*^p < .05; ^**^p < .01; ^***^p < .001.

Models adjusting for psychological distress at 1 month postpartum explained 14–19% of the variance of mother-infant bonding at 3 months postpartum. For the intrusion and hyperarousal subscales, as with the full scale, when maternal general psychological distress at 1 month postpartum was added to the model, it showed to be significantly prospectively associated with mother infant bonding at 3 months postpartum (intrusions: *B* = .01, *β* = .27, *p* = .011, 95% CI = [.003,.02]; hyperarousal: *B* = .04, *β* = .05, *p* = .642, 95% CI = [.01,.04]). The changes adjusting for concurrent maternal psychological distress created in the models suggest that concurrent maternal psychological distress is implicated in the association between maternal PTSD-CB symptoms at 1 month postpartum and mother-infant bonding at 3 months postpartum. Therefore, mediation models were run to further investigate the nature of this role. Models examining the mediation of the relationship between maternal symptom subscales at 1 month postpartum and mother-infant bonding at 3 months postpartum explained 17% to 19% of the variance of mother-infant bonding at 3 months postpartum. Interestingly, a significant indirect effect, suggestive of mediation of the association between maternal PTSD-CB symptoms and mother-infant bonding by maternal general psychological distress was found for the intrusion and hyperarousal subscales (see **Figures 5A, B**). For both, intrusions and hyperarousal, higher symptom scores on the subscales at 1 month postpartum were associated with higher psychological distress at 1 month postpartum, which in turn was prospectively associated to worse mother-infant bonding at 3 months postpartum. However, there was no total effect found in either model (intrusions: *B* = .10, *β* = .18, *p* = .266, 95% CI = [-.08,.28]; hyperarousal: *B* = .03, *β* = .50, *p* = .644, 95% CI = [-.12,.20]). The mediation model for the maternal intrusion symptoms subscale explained 16% of the variance of mother-infant bonding at 3 months postpartum, while the mediation model for the hyperarousal symptom subscale explained 17% of the variance of mother-infant bonding at 3 months postpartum.

#### Fathers

All the paternal symptom subscales showed the same pattern as the full symptom scale when assessing the prospective association between paternal PTSD-CB symptoms at 1 month postpartum and father-infant bonding at 3 months postpartum. These models using the symptom subscales explained 8% to 22% of the variance of father-infant bonding at 3 months postpartum. After adjusting for paternal general psychological distress at 1 month postpartum, the model for the paternal avoidance symptom subscale was the same as for the full symptom scale: no pathways remained significant. For the paternal intrusion symptom subscale, the pathway between paternal general psychological distress at 1 month postpartum and father-infant bonding at 3 months postpartum was the only significant pathway (*B* = .02, *β* = .46, *p* = .019, 95% CI = [.004,.04]). For the paternal hyperarousal symptom subscale, the pathway between paternal general psychological distress at 1 month postpartum and paternal hyperarousal symptoms remained significant (*B* = .03, *β* = .42, *p* = .007, 95% CI = [.01,.06]). The subscale symptom models adjusting for paternal psychological distress at 1 month postpartum accounted for 19–31% of the variance of father-infant bonding at 3 months postpartum. Once again, given the lack of impact testing paternal concurrent psychological distress as a confounder has had on the model, we were not justified to test concurrent psychological distress as a mediator for fathers.

## Discussion

This prospective population-based cohort study investigated the prospective relationships between parental PTSD-CB symptoms at 1 month postpartum and parent-infant bonding at 3 months postpartum, whilst adjusting for antenatal factors. Furthermore, we examined whether concurrent psychological distress was implicated in the relationship between PTSD-CB symptoms at 1 month postpartum and parent-infant bonding at 3 months postpartum first by adjusting for psychological distress at 1 month postpartum and then by testing whether concurrent psychological distress acted as a mediator. These investigations were carried out separately for mothers and fathers.

In mothers, as hypothesized, we found evidence that maternal PTSD-CB symptoms at 1 month postpartum were negatively prospectively associated with mother-infant bonding at 3 months postpartum, after adjusting for antenatal factors. This effect was also seen for mothers in the avoidance symptom subscale. However, no such association was seen for fathers. Interestingly, we also found an association between higher paternal PTSD-CB symptoms at 1 month postpartum and mother-infant bonding at 3 months postpartum with a small effect, but no such cross-association between maternal PTSD-CB symptoms at 1 month postpartum and father-infant bonding at 3 months postpartum. This result may suggest that parents’ PTSD-CB symptoms at 1 month postpartum and parent-infant bonding are differentially associated and should not be assumed to be similar. However, it should be noted that due to small sample size antenatal factors were not adjusted for in this analysis and such cross-associations require further investigation before conclusions are drawn. Therefore, overall, we found partial support for our hypothesis that PTSD-CB symptoms at 1 month postpartum would be negatively prospectively associated with parent-infant bonding at 3 months postpartum. Once we adjusted for psychological distress at 1 month postpartum, the prospective association between maternal PTSD-CB symptoms at 1 month postpartum and mother-infant bonding at 3 months postpartum disappeared. The same pattern was also seen in the maternal avoidance symptom subscale. From this exploratory investigation, we found no evidence that the prospective association between PTSD-CB symptoms at 1 month postpartum and mother-infant bonding at 3 months postpartum remains after adjusting for psychological distress at 1 month postpartum. Conversely, this provides evidence that concurrent maternal psychological distress appears to have a role in the association between maternal PTSD-CB symptoms at 1 month postpartum and mother-infant-bonding at 3 months postpartum. This led us to investigate further the nature of this role by assessing concurrent maternal psychological distress as a mediator. For mothers, evidence for a mediation between PTSD-CB symptoms at 1 month postpartum and mother-infant bonding at 3 months postpartum by psychological distress at 1 month postpartum was found only for the intrusion and hyperarousal symptom subscales at 1 month postpartum. Here, there was a significant indirect effect where both maternal symptoms of intrusions and hyperarousal at 1 month postpartum were associated with higher maternal psychological distress at 1 month postpartum, which was prospectively associated with worse mother-infant bonding at 3 months postpartum. No such evidence was found for the full scale of maternal PTSD-CB symptoms at 1 month postpartum. This investigation could not be conducted for fathers given the lack of effect adjusting for concurrent paternal psychological distress had on the models for fathers which meant that further investigation using mediation was not justified for the full scale or any of the subscales. Thus, the question of the mechanistic relationships, if any, between paternal psychological distress, paternal PTSD symptoms, and father-infant bonding remains untested.

ur result that maternal PTSD-CB symptoms at 1 month postpartum had a negative prospective association with mother-infant bonding at 3 months postpartum is consistent with previous cross-sectional literature investigating early parent-infant relationships. Dekel et al. (18) found that probable PTSD-CB predicted less maternal attachment assessed within 6 months postpartum, after adjusting for other perinatal factors. Also, within the first 6 weeks postpartum Davis et al. (22) found that mothers who were fully symptomatic or partially symptomatic with regards to PTSD-CB had a more negative representation of their infant and a less optimal maternal attachment, compared to those who were not symptomatic. These results reflect effects before depressive symptoms were taken into account. Thus, our results add to this body of evidence.

To our knowledge, no previous study has looked at the prospective relationship between paternal PTSD-CB symptoms and father-infant bonding in the early postpartum. While we found no prospective association between paternal PTSD-CB symptoms at 1 month postpartum and father-infant bonding at 3 months postpartum, the standardized coefficient between PTSD-CB symptoms and paternal bonding is the largest in the model. This could indicate that with an increase in statistical power, this result may have become significant, but replication with an even larger sample would be required to substantiate this.

Results concerning the relationship between PTSD-CB and maternal-infant bonding do remain inconsistent (19) and this partly depends on whether depression had been taken into account. Once psychological distress at 1 month postpartum was added to the model, the predictive relationship between maternal PTSD-CB symptoms at 1 month postpartum and mother-infant bonding at 3 months postpartum disappeared. However, the predictive relationship between maternal psychological distress at 1 month postpartum and mother-infant bonding at 3 months postpartum was significant. This result may point to maternal psychological distress at 1 month postpartum being more important than maternal PTSD-CB symptoms at 1 month postpartum for maternal-infant bonding at 3 months postpartum. Our results are in line with one study showing that the result of mothers with full or partial symptoms of PTSD-CB having a more negative representation of their infant and a less optimal maternal-infant attachment than those who were not symptomatic, disappeared once depressive symptoms were adjusted for (22). The difference between those fully/partially symptomatic and not symptomatic groups only remained for the maternal perception of infants’ emotional warmth. Our result is, however, in contrast with other studies. One cross-sectional study found that even when depressive symptoms were included in the model, maternal PTSD-CB symptoms did predict mother-infant bonding (20). However, this study used depressive symptoms rather than psychological distress, did not necessarily aim to adjust for depressive symptoms in the relationship between CB-PTSD symptoms and mother-infant bonding, and did not adjust for antenatal variables. The authors did note that the respective effects between mother-infant bonding and PTSD-CB symptoms as well as mother-infant bonding and depressive symptoms were similar and that the overlap between the symptoms should be further considered. Only one study looked prospectively at predictors of maternal-infant bonding at 15 months postpartum, but did not find any significant prediction by maternal PTSD-CB symptoms or psychological distress symptoms at 3 months postpartum (10). However, it should be noted that this study investigated a different time period, did not adjust for antenatal factors, and was not as well powered as our study. Our study specifically focused on the early postpartum period because a better understanding of the mechanisms during this period will help to inform early intervention strategies. Finally, it is possible that different mechanisms may be at work during the early vs. later postpartum period. For example, infant variables, such as infant temperament, may play a less important role shortly after birth compared to later on.

Regarding fathers, the only change adding paternal psychological distress at 1 month postpartum was that the standardized coefficient between paternal PTSD-CB at 1 month postpartum and father-infant bonding at 3 months postpartum was no longer the largest in the model. A decrease in the coefficient between parental PTSD-CB at 1 month postpartum and parent-infant bonding at 3 months postpartum when adding psychological distress at 1 month postpartum was also the case amongst the maternal models, and when the subscales were investigated. Unlike for mothers, psychological distress at 1 month postpartum was not a significant predictor of father-infant bonding at 3 months postpartum, regardless of whether psychological distress at 1 month postpartum was included. One other study has investigated the predictors of father-infant bonding at 15 months postpartum and found no prediction by paternal PTSD symptoms at 3 months postpartum, although psychological distress symptoms at 3 months postpartum was a significant predictor (10). As for mothers, this latter study indicated a different pattern of results than those found here, but also assessed parent-infant bonding at 15 months postpartum. It may be that this points to a dynamic relationship between parental mental health variables and parent-infant bonding over time in the postpartum period. The latter study (10) also over sampled those with symptoms of PTSD-CB and depression, indicating their study may have looked at more “clinical” parents than ours. These points suggest that further studies would be warranted that investigate prospective relationships in both a community and a clinical sample over different time points postpartum.

Only one other study has investigated the mechanistic relationships of maternal postpartum affective symptoms (depression/anxiety/psychological distress), PTSD-CB, and mother-infant bonding within the first year postpartum. This study found that the relationship between PTSD-CB symptoms and mother-infant bonding was mediated by depressive symptoms (26). This is in contrast to our findings of a lack of a mediation between maternal PTSD-CB symptoms and mother-infant bonding by maternal psychological distress. Having said this, we do find evidence of mediation when using the intrusion symptoms subscale and hyperarousal symptoms subscale, so our results are not in complete contrast to Williams et al. (26), rather they are more nuanced. There are several important differences between this study and that of Williams et al. (26) which may have contributed to the difference in results between studies. Our study assesses prospective relationships rather than cross-sectional relationships, we adjusted for antenatal factors, and assessed psychological distress, rather than depressive symptoms. Also, Williams et al. (26) oversampled those with depressive and PTSD-CB symptoms, then applied cut-offs, suggesting that their sample reflects a more “clinical” sample than ours and their results reflect relationships with probable PTSD rather than PTSD symptoms severity, as in our study.

Our results from assessing each research question with the intrusion, hyperarousal, and avoidance symptoms subscales indicate that there are some nuances amongst the relationships between parental PTSD-CB symptoms at 1 month postpartum, parental psychological distress at 1 month postpartum, and parent-infant bonding at 3 months postpartum. The analyses with the subscales allowed us to see if there was a particular symptom cluster that may be driving the effects seen with the full scale. For example, amongst the subscales, only maternal avoidance symptoms at 1 month postpartum showed a negative prospective association with mother-infant bonding at 3 months postpartum. If the mother reported symptoms of avoidance, then it is likely that she actively avoided interactions with her child. This would make it more difficult for a mother to develop positive feelings towards her child or learn to read the child’s signals and thus understand the child’s needs. The mediation for mothers was found for the intrusion and hyperarousal symptom subscales. Such results may indicate that there is actually a complex relationship between the three variables. Future studies would benefit from a measurement of parental psychological distress at a time point in-between parental PTSD-CB symptoms and parent-infant bonding to fully assess the mediation question and consideration of subscales within analyses may help to provide insight into the relationships between the three variables over the postpartum period.

It is interesting to note that our results display different patterns of relationships between parental PTSD-CB symptoms at 1 month postpartum and parent-infant bonding at 3 months postpartum for mothers and fathers. There appears to be more evidence for a prospective association for parental PTSD-CB symptoms for mothers than fathers, although across models more variance is explained on parent-infant bonding for fathers than for mothers. Thus, while the mental health variables identified here may not be showing a significant association, the full structural equation model seems to be a good estimation of potential important variables associated with father-infant bonding at 3 months postpartum. It may also indicate, as previously pointed out, that if the sample of fathers was more powered, we may have seen more evidence of associations between paternal mental health variables at 1 month postpartum and father-infant bonding at 3 months postpartum.

When considering what may be making the differences in results for mothers and fathers, it is important to consider the time-frame that our results reflect. Our results only show the prospective association of PTSD-CB symptoms at 1 month postpartum with parent-infant bonding at 3 months postpartum. It may be that 3 months postpartum is too early to see an effect of PTSD-CB symptoms on father-infant bonding. By 3 months postpartum, Swiss fathers are likely to be at work and thus have less time to spend with their infant, with their daily focus not necessarily being tied up in their role as a father. Under this rationale, at this early point, it may be that mental health variables are affecting other areas of their life e.g., work, rather than factors related to their child. We should also keep in mind the point about the time-frame discussed above, i.e., we cannot rule out that paternal mental health variables may show an association with father-infant bonding later on in time. For mothers, it seems that we may be already starting to see some associations with maternal PTSD-CB symptoms and psychological distress symptoms at this 3 months postpartum point. Swiss mothers are likely to be at home on maternity leave, thus their daily role at this moment is likely caring for their infant and likely focused on their infant and their relationship with their infant, with many opportunities for interactions. Therefore, it makes intuitive sense that mental health variables may be influencing variables related to their mother-child relationship. Furthermore, as mothers often spend more time with their infants than fathers, they may have more opportunities to test their expectations regarding their infant. Some may find that their expectations have not been fully realized, thus altering their feelings towards their child. These suggestions would require testing with further research, ideally taking factors around paternal occupation and maternity leave, as well as parental expectations towards their infant into account.

Concerning the clinical implications of our results, our results indicate that maternal but not paternal mental health symptoms are already having some impact on the mother-infant relationship by 3 months postpartum. It seems likely that maternal psychological distress is responsible for the larger part, however, mediation results imply that intrusion and hyperarousal symptoms are also relevant for this relationship. Therefore, early interventions for reducing maternal PTSD-CB or psychological distress symptoms may prevent problems in bonding with the infant. We do not know, however, if effects of parental mental health symptoms will get stronger if symptoms are not treated nor if the impact on parental-infant bond will improve with symptom reduction or whether a targeted intervention is required. We should point out that we cannot generalize our findings to those parents with clinically relevant symptoms of PTSD-CB or psychological distress (anxiety/depression), thus it would be of interest for future studies to assess these questions with a clinical sample.

This study inevitably has some limitations. Several concern the cohort from which this sample was taken. Participants were recruited *via* convenience sampling. It maybe that those who were interested in psychological well-being were more likely to take part. Furthermore, only French speakers were included in a population where English, German, and Italian are also spoken, which would more likely exclude those with a migrant or refugee status. These two points may limit the generalizability of the findings. Indeed, as reported above, the sample was reported as mainly Swiss, university educated, living as a cohabiting or married couple. This study is also reliant on self-report questionnaires and results should be interpreted within the limits of self-report data, e.g., associations refer to the perception of symptoms and may be subject to some social desirability. Finally, it should be noted that PTSD-CB symptoms relate to PDS-F for DSM-IV, as this was the valid assessment tool when data collection for this cohort commenced.

Another limitation is that the models for fathers appeared to suffer from a lack of statistical power. Given the amount of variance explained and size of some standardized coefficients, it is surprising that more pathways did not appear as significant. In this study we were not justified to run the mediation models for fathers; therefore, this question remains uninvestigated for fathers. Also, given the size of some of the standardized coefficients in the models for mothers, it may well be that other pathways would become significant with an increase in statistical power. Ideally, for assessing the question of mediation, we would have had a time point in-between parental PTSD-CB symptoms and parent-infant bonding where psychological distress would have been measured. We did attempt to reduce the risk of bias within the mediation analysis from having concurrent parental PTSD-CB symptoms and psychological distress symptoms by adjusting carefully for antenatal PTSD symptoms and antenatal psychological distress, as suggested by Cole and Maxwell (39). Future studies wishing to look at the temporal, mechanistic relations between PTSD symptoms, psychological distress, and parent infant bonding would need to measure psychological distress at an intermediate time point.

This study also benefits from certain strengths. The prospective design allowed relationships between variables to be investigated over time and, by adjusting for antenatal factors, this study was able to ensure that only relationships between variables **postpartum** were estimated. This study also included fathers, who are an integral part of the parenting team and any intergenerational transmission of stress and trauma occurring through the postpartum environment could also occur through the father, as well as the mother (5). Through use of a community sample, we were able to see whether PTSD-CB symptoms found amongst the general population were related to parent-infant bonding. Not only can this give us an idea of whether such relationships would be worth further consideration in a clinical sample, but also highlights that at a community level, we should be aware of mental health variables and when prevention/interventions may be appropriate. The final strength of this study is that some previous literature has assessed the specific relationship amongst PTSD-CB symptoms, *depressive* symptoms and parent-infant bonding. However, we assessed psychological distress, which incorporates *both anxiety and depression*. Future studies may wish to specifically investigate the temporal mechanistic relationships between symptoms of postpartum depression, PTSD-CB, and parent-infant bonding, but it is clear from our results that it is not only symptoms of postpartum depression that seem to be playing a role in how parental mental health variables and parent-infant bonding are related.

## Conclusions

This investigation of the prospective relationship between parental PTSD-CB symptoms at 1 month postpartum and parent-infant bonding at 3 months postpartum after adjusting for antenatal factors and considering the role of parental psychological distress at 1 month postpartum indicates that mothers and fathers do not evidence the same effects. For mothers, the negative prospective association between PTSD-CB symptoms at 1 month postpartum and mother-infant bonding at 3 months postpartum disappears after adjusting for psychological distress at 1 month postpartum. Thus, implicating maternal concurrent psychological distress in the association between maternal PTSD-CB symptoms at 1 month postpartum and mother-infant bonding at 3 months postpartum. Evidence for a mediation of maternal PTSD-CB symptoms at 1 month postpartum and mother-infant bonding at 3 months postpartum by maternal psychological distress at 1 month postpartum was seen in the intrusion and hyperarousal symptom subscale. These results indicate certain nuances exist in the relationship between PTSD-CB symptoms, psychological distress, and mother-infant bonding. No such evidence of a prospective association of PTSD-CB symptoms or psychological distress symptoms was found for fathers. However, the complete structural equation models did explain more variance on father-infant bonding for fathers than mothers and the size of coefficients in the models suggest with greater power more associations may have been significant. Therefore, these results should not deter further investigation in the potential prospective association of paternal mental health with father-infant bonding. Rather more research is required to complete the mechanistic investigation and look longer term into the first postpartum year. Results may indicate early intervention on paternal PTSD-CB symptoms and mother-infant bonding may be appropriate to mitigate potential negative effects of parental mental health on parent-infant bonding. Results with a clinical sample and at different time points across the postpartum period would assist in further scrutiny of this proposal.

## Data Availability Statement

The raw data supporting the conclusions of this article will be made available by the authors, without undue reservation.

## Ethics Statement

The studies involving human participants were reviewed and approved by Commission cantonale d’éthique de la recherche sur l’être humain. The patients/participants provided their written informed consent to participate in this study.

## Author Contributions

This study was conceived, research questions developed, and the conceptual model created by AH, SG-N, and SS. AH conducted the literature search. SG-N and SS developed the analysis plan in consultation with AH, which SS conducted. Data were interpreted by AH, SG-N, and SS. AH, SG-N, and SS wrote the manuscript. AH designed the data collection instruments, coordinated, and supervised data collection. All authors contributed to the article and approved the submitted version.

## Funding

No funding was provided for this work but AH and SG-N are management committee members of COST action CA18211.

## Conflict of Interest

The authors declare that the research was conducted in the absence of any commercial or financial relationships that could be construed as a potential conflict of interest.
