# Supplementary material for: Parental Birth-Related PTSD Symptoms and Bonding in the Early Postpartum Period: A Prospective Population-Based Cohort Study
Source: Front Psychiatry. 2020 Sep 23;11:570727. doi: 10.3389/fpsyt.2020.570727 (PMC7540215; doi:10.3389/fpsyt.2020.570727)
Supplement: Supplementary file 1 [file DataSheet_1.docx]

**Supplementary materials**


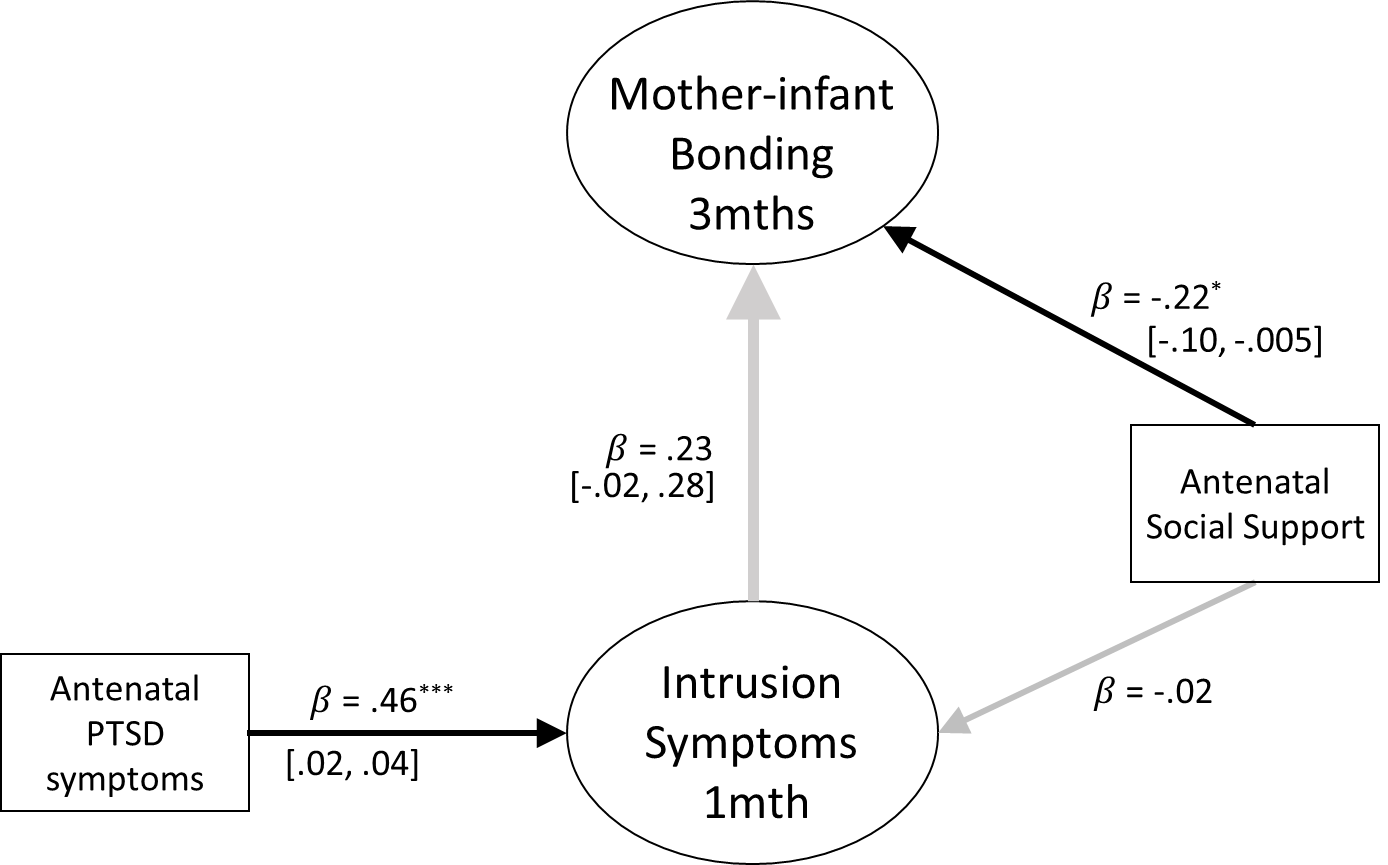


Figure 1. Path model of the prospective prediction of **mother**-infant bonding at 3 months by intrusion symptoms at 1 month. Antenatal social support and PTSD symptoms are included as covariates. Black lines indicate significant pathways, grey lines indicate non-significant pathways. Standardised coefficients and 95% confidence intervals are reported. ^*^p < .05; ^**^p < .01; ^***^p <.001. Accounted for 12% variance on mother-infant bonding.


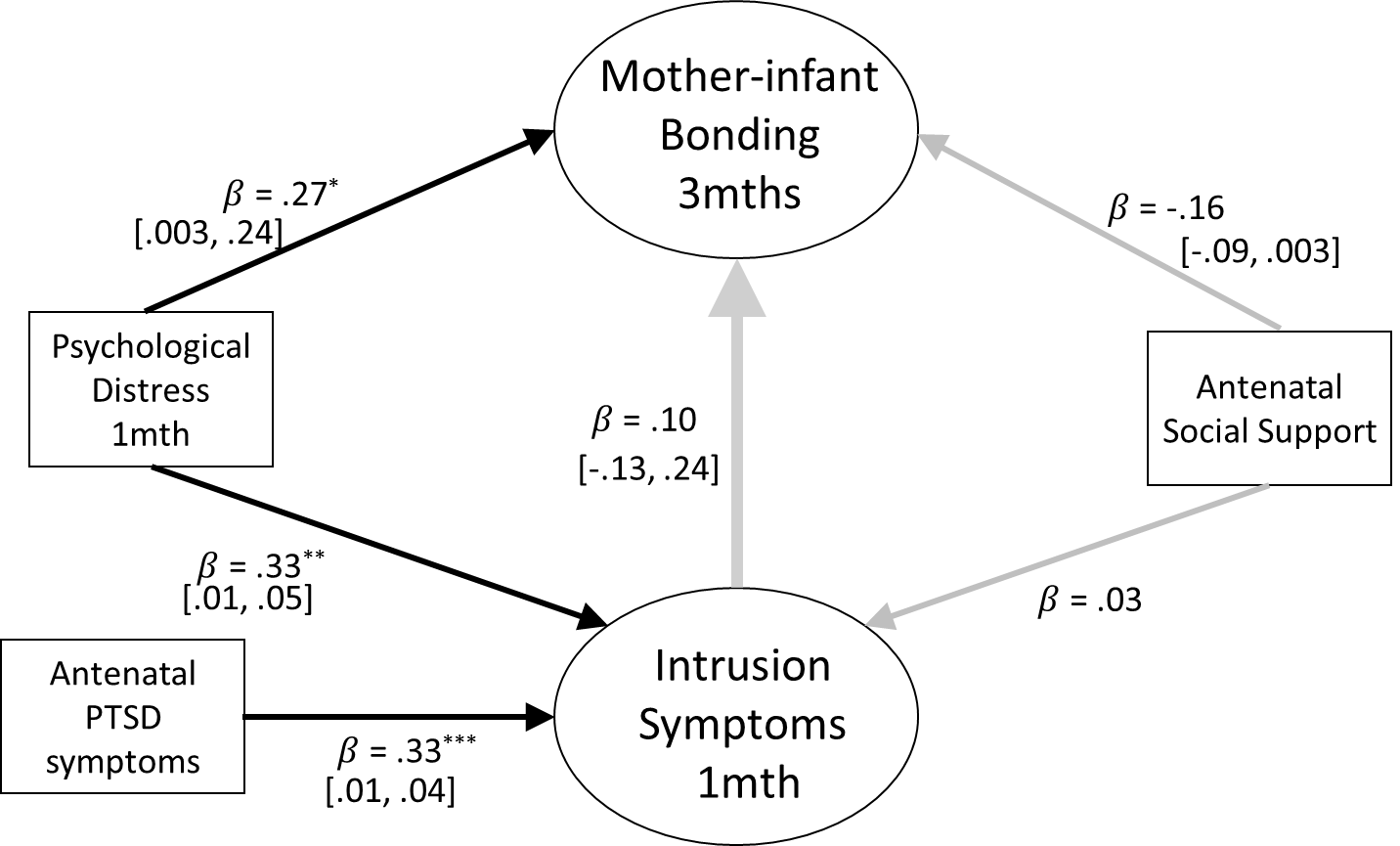


Figure 2. Path model of the prospective prediction of **mother**-infant bonding at 3 months by intrusion symptoms at 1 month controlling for concurrent psychological distress . Antenatal social support and PTSD symptoms are also included as covariates. Black lines indicate significant pathways, grey lines indicate non-significant pathways. Standardised coefficients and 95% confidence intervals are reported. ^*^p < .05; ^**^p < .01; ^***^p <.001. Accounted for 17% of the variance on mother-infant bonding.


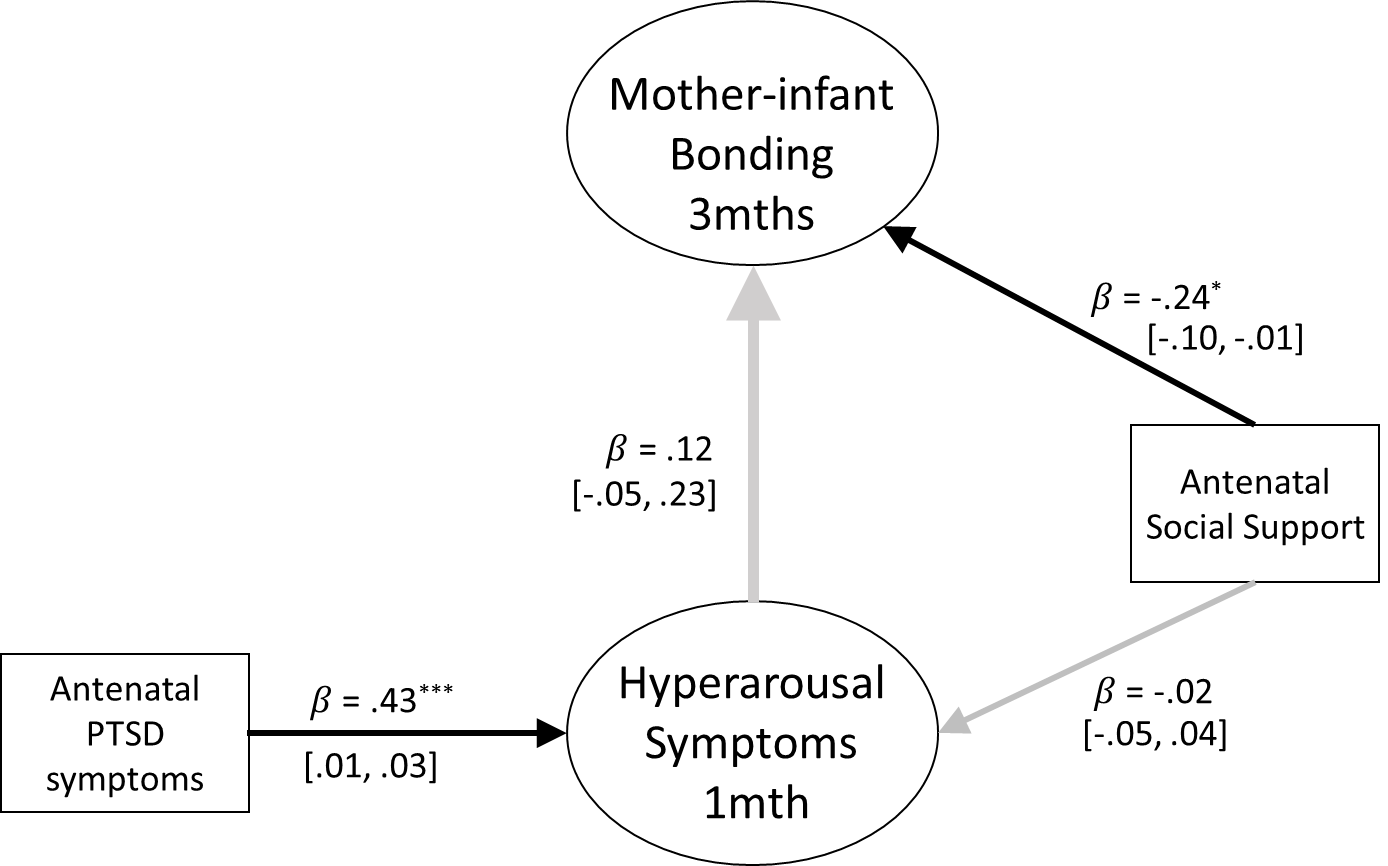


Figure 3. Path model of the prospective prediction of **mother**-infant bonding at 3 months by hyperarousal at 1 month. Antenatal social support and PTSD symptoms are included as covariates. Black lines indicate significant pathways, grey lines indicate non-significant pathways. Standardised coefficients and 95% confidence intervals are reported. ^*^p < .05; ^**^p < .01; ^***^p <.001. Accounted for 19% variance on mother-infant bonding. Accounted for 8% of the variance on mother-infant bonding.


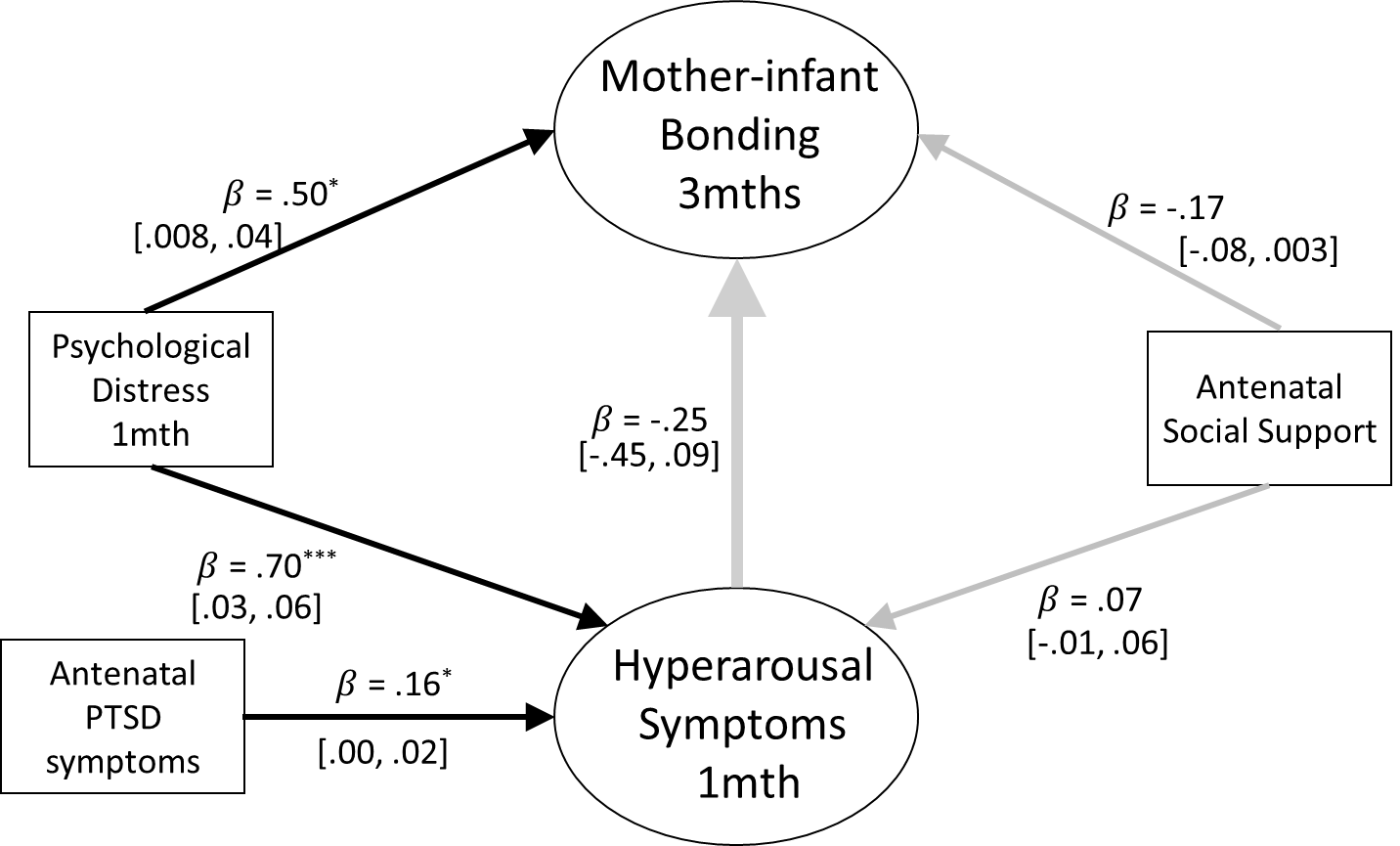


Figure 4. Path model of the prospective prediction of **mother**-infant bonding at 3 months by hyperarousal symptoms at 1 month controlling for concurrent psychological distress . Antenatal social support and PTSD symptoms are also included as covariates. Black lines indicate significant pathways, grey lines indicate non-significant pathways. Standardised coefficients and 95% confidence intervals are reported. ^*^p < .05; ^**^p < .01; ^***^p <.001. Accounted for 18% of the variance on mother-infant bonding.


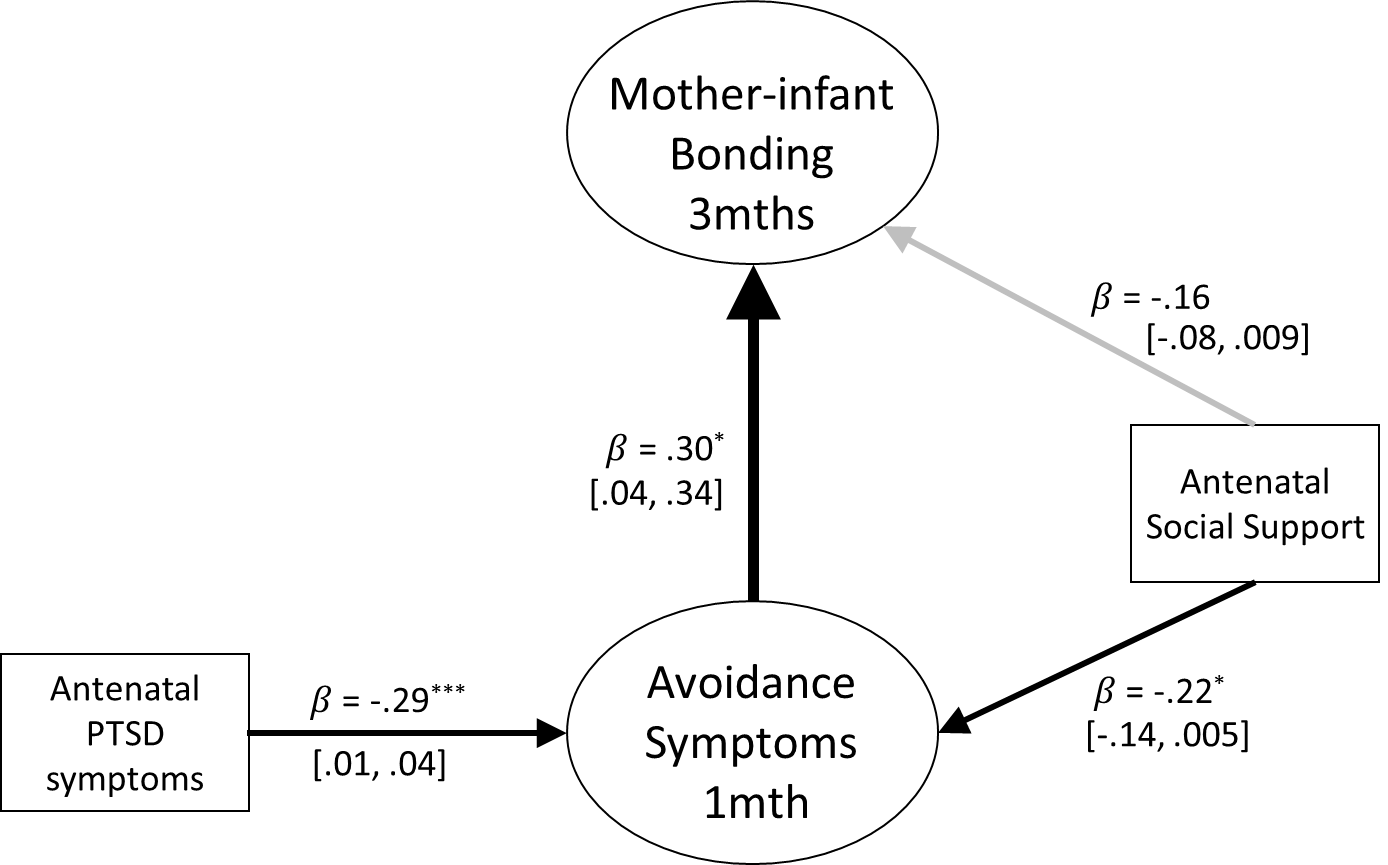


Figure 5. Path model of the prospective prediction of **mother**-infant bonding at 3 months by intrusion symptoms at 1 month. Antenatal social support and PTSD symptoms are included as covariates. Black lines indicate significant pathways, grey lines indicate non-significant pathways. Standardised coefficients and 95% confidence intervals are reported. ^*^p < .05; ^**^p < .01; ^***^p <.001. Accounts for 15% of variance on bonding.


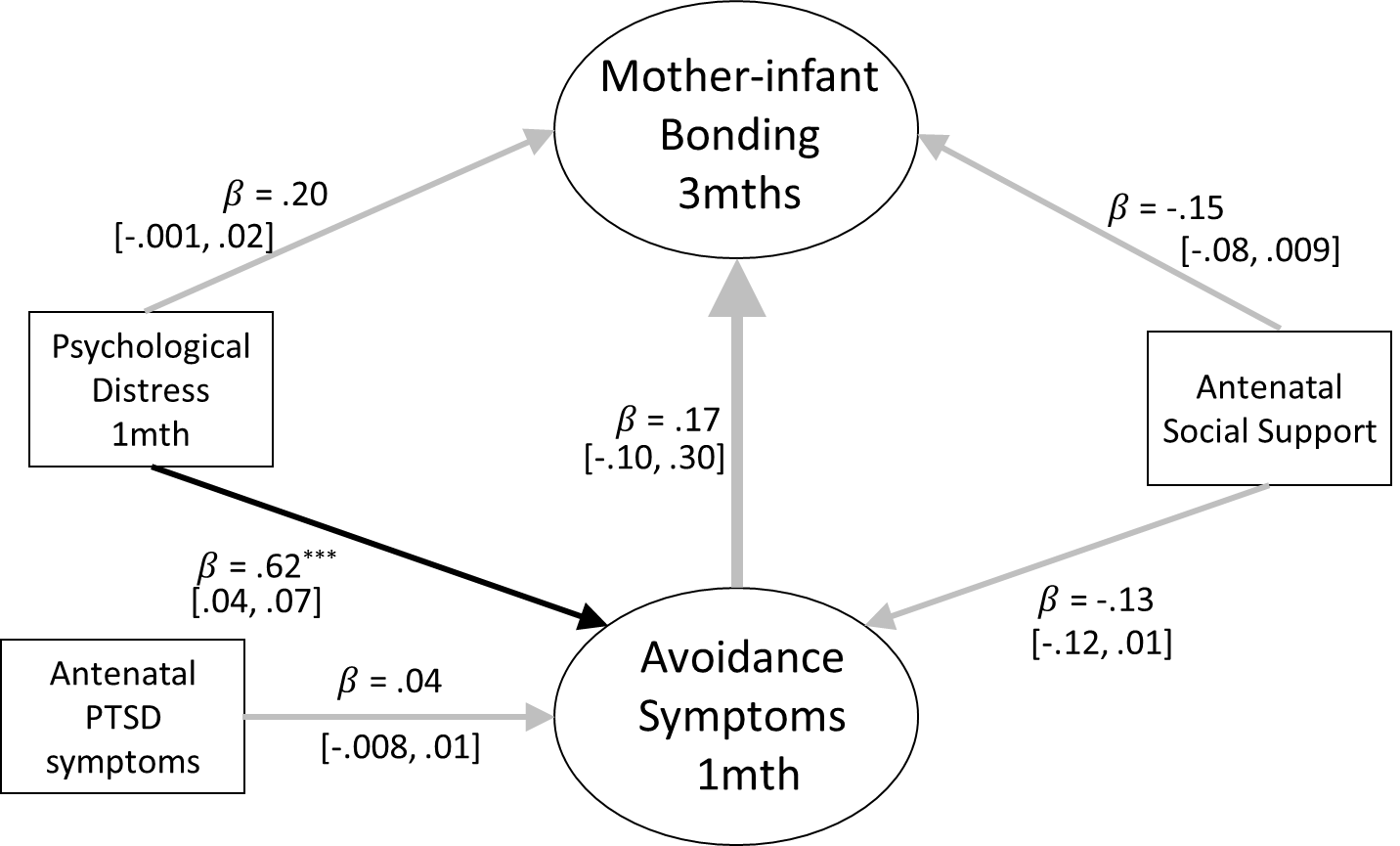


Figure 6. Path model of the prospective prediction of **mother**-infant bonding at 3 months by avoidance symptoms at 1 month controlling for concurrent psychological distress . Antenatal social support and PTSD symptoms are also included as covariates. Black lines indicate significant pathways, grey lines indicate non-significant pathways. Standardised coefficients and 95% confidence intervals are reported. ^*^p < .05; ^**^p < .01; ^***^p <.001.


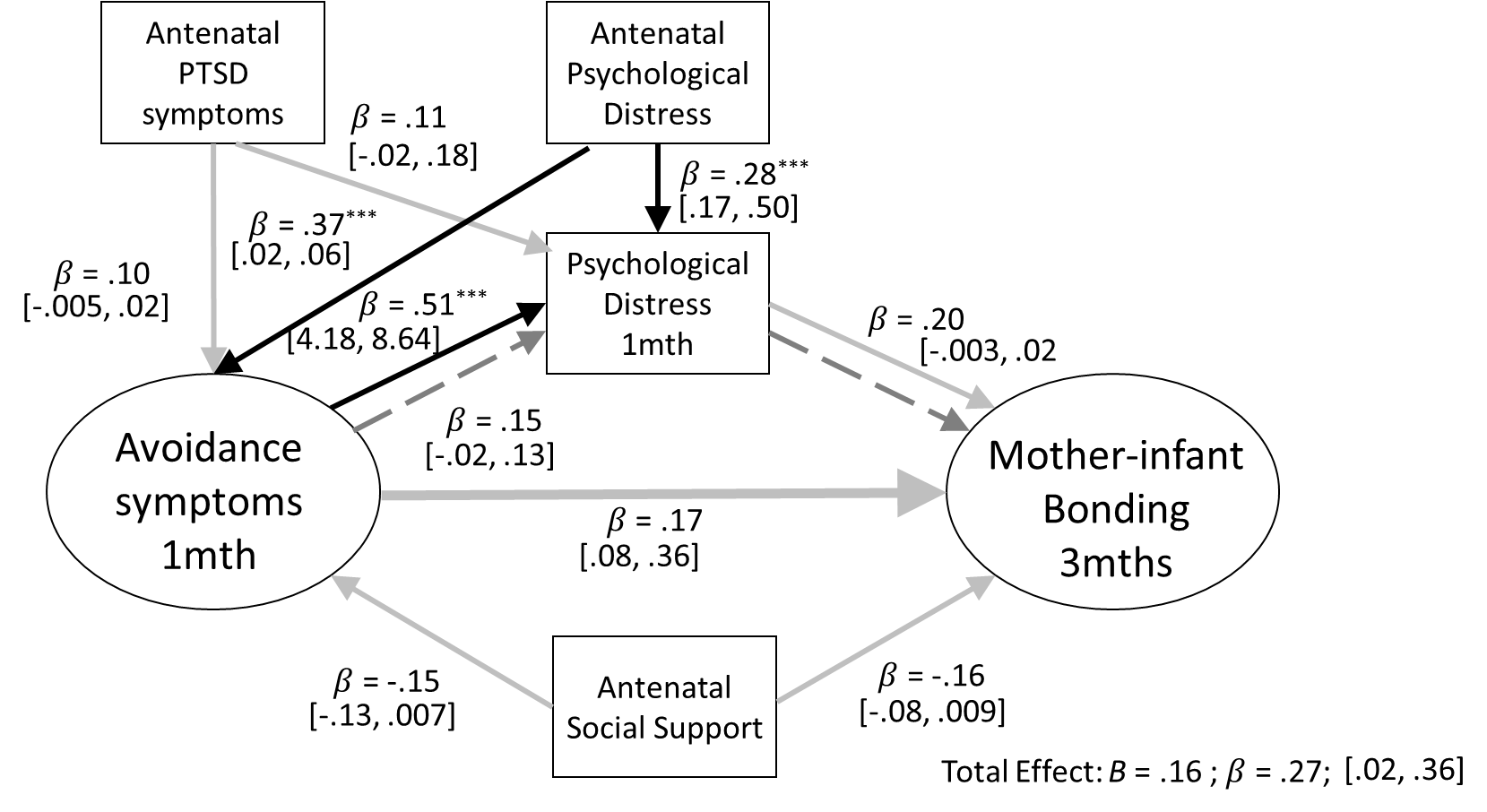


Figure 7. Path model of the mediation of the relationship between **mother**-infant bonding at 3 months and avoidance symptoms at 1 month by concurrent psychological distress . Antenatal social support and PTSD symptoms are also included as covariates. Black lines indicate significant pathways, grey lines indicate non-significant pathways, dashed lines signfiy the indirect effect. Standardised coefficients and 95% confidence intervals are reported. ppPTSD = postpartum PTSD symptoms. ^*^p < .05; ^**^p < .01; ^***^p <.001. This model accounted for 17% of variance on mother-infant bonding.


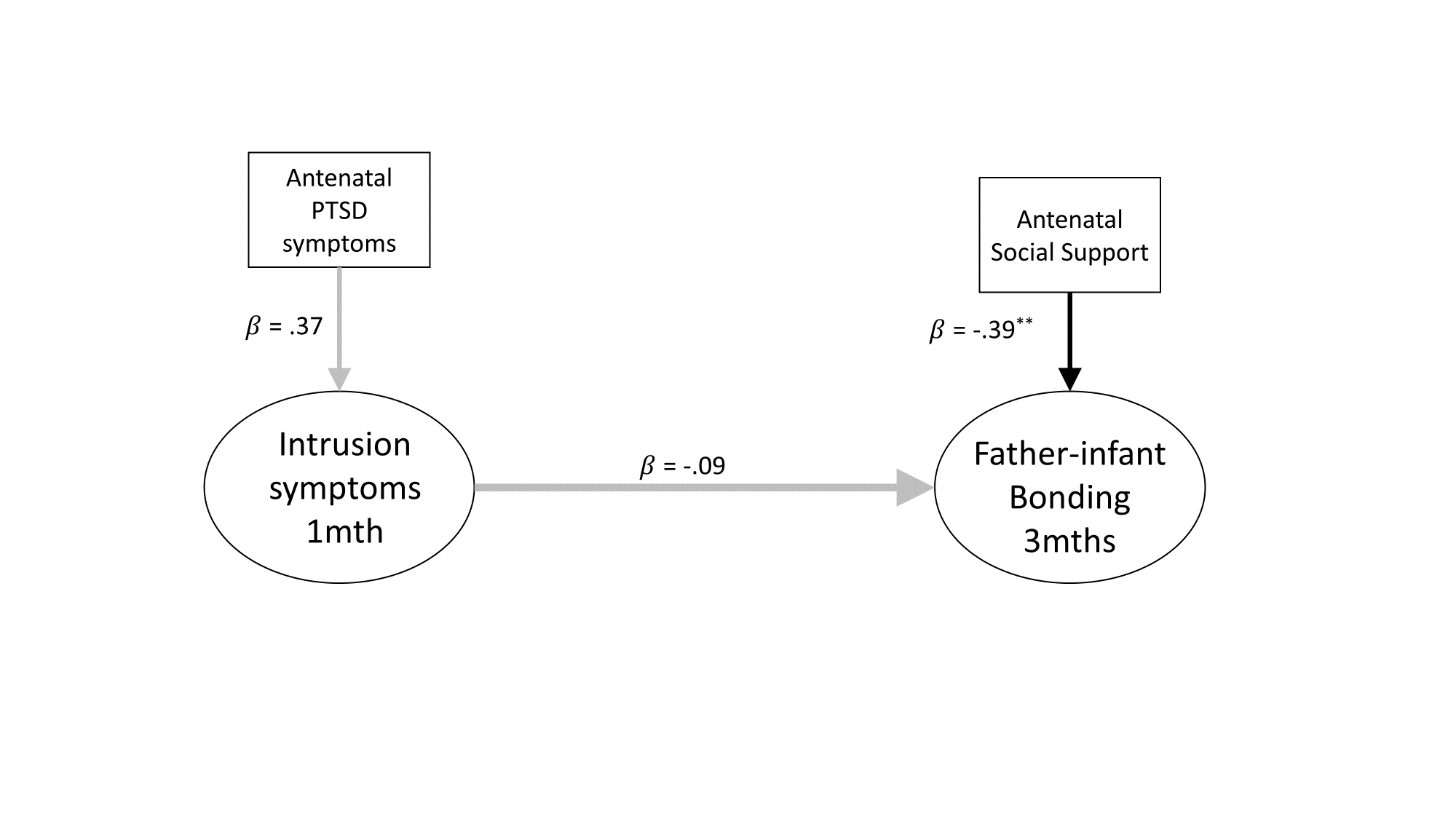


Figure 8. Path model of the prospective prediction of **father**-infant bonding at 3 months by intrusion symptoms at 1 month. Antenatal social support and PTSD symptoms are included as covariates. Black lines indicate significant pathways, grey lines indicate non-significant pathways. Standardised coefficients and 95% confidence intervals are reported. ^*^p < .05; ^**^p < .01; ^***^p <.001. Accounted for 15% of the variance on father-infant bonding.


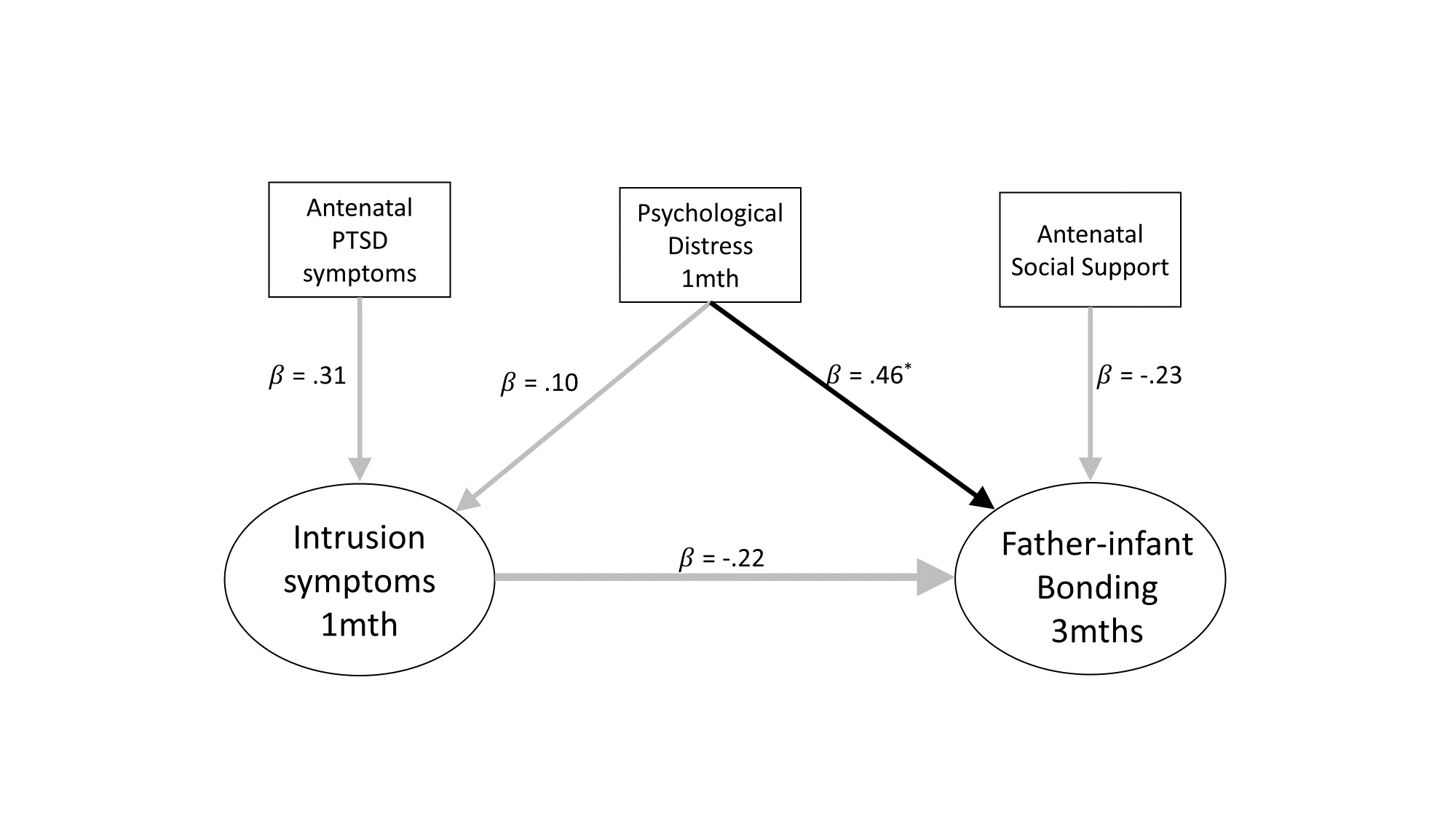


Figure 9. Path model of the prospective prediction of **father**-infant bonding at 3 months by intrusions symptoms at 1 month controlling for concurrent psychological distress . Antenatal social support and PTSD symptoms are also included as covariates. Black lines indicate significant pathways, grey lines indicate non-significant pathways. Standardised coefficients and 95% confidence intervals are reported. ^*^p < .05; ^**^p < .01; ^***^p <.001. Accounted for 31% of the variance on father-infant bonding. Accounted for 31% of variance on father-infant bonding.


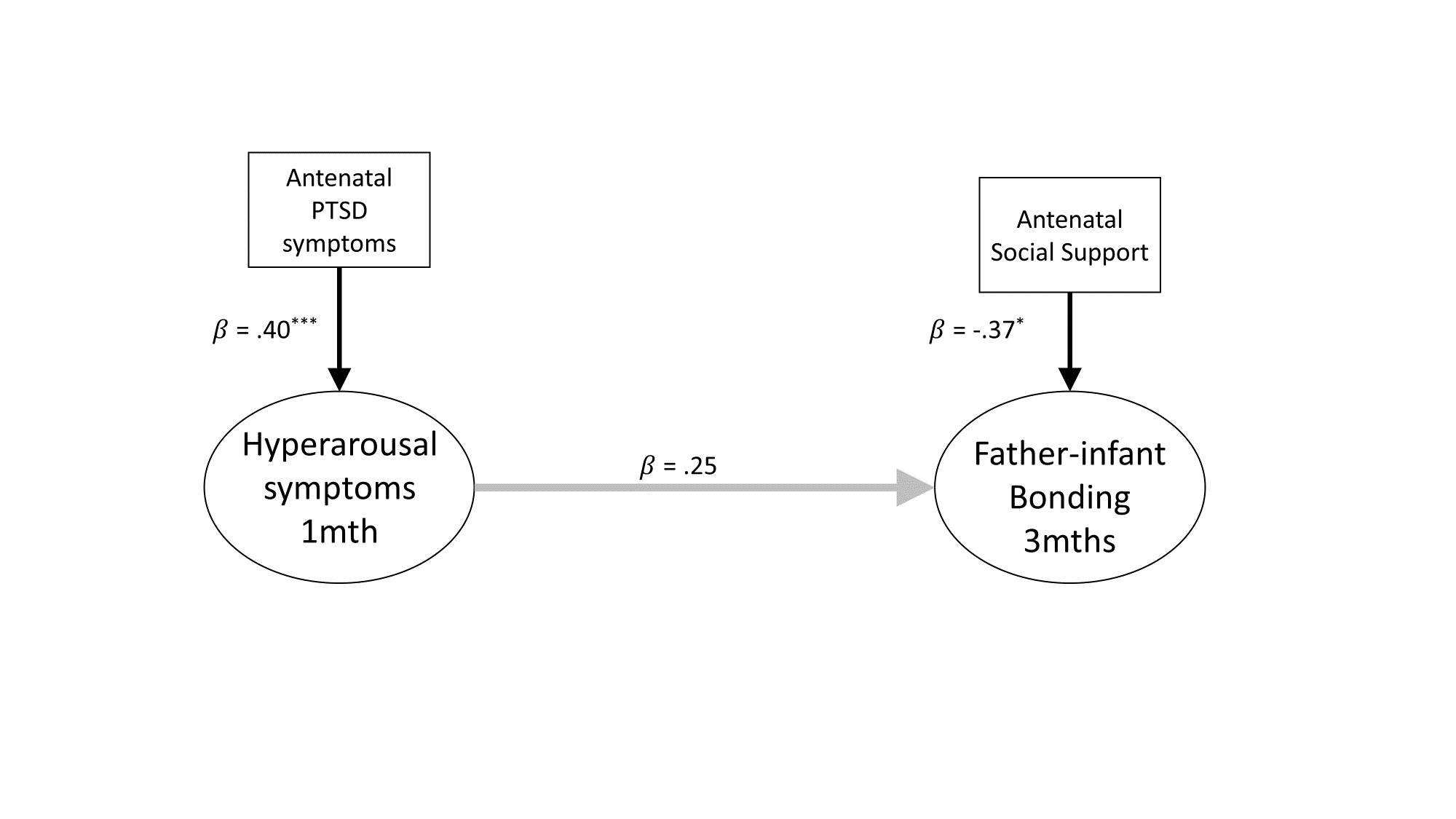


Figure 10. Path model of the prospective prediction of **father**-infant bonding at 3 months by hyperarousal symptoms at 1 month. Antenatal social support and PTSD symptoms are included as covariates. Black lines indicate significant pathways, grey lines indicate non-significant pathways. Standardised coefficients and 95% confidence intervals are reported.^*^p < .05; ^**^p < .01; ^***^p <.001. Accounted for 22% of the variance on father-infant bonding.


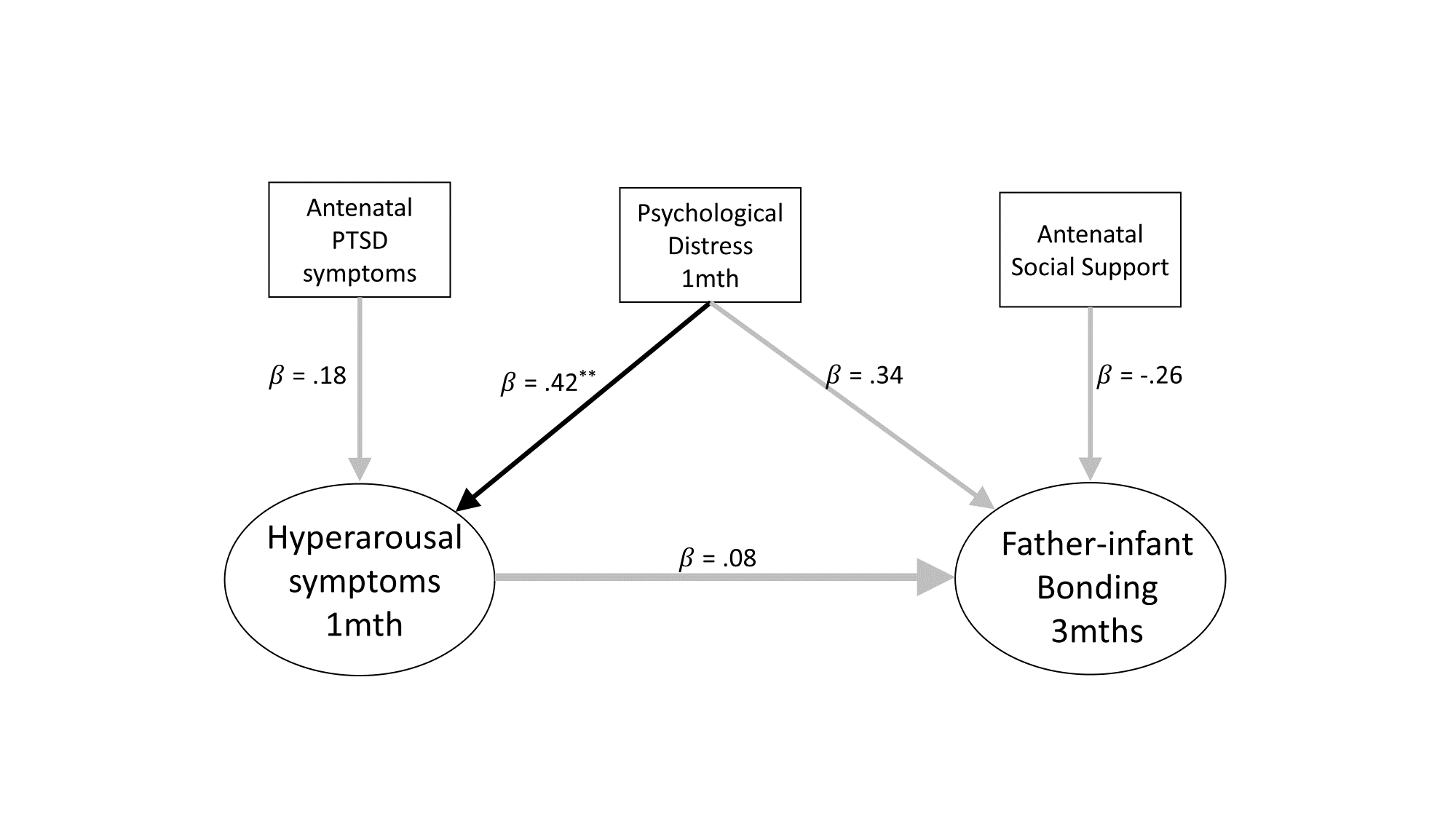


Figure 11. Path model of the prospective prediction of **father**-infant bonding at 3 months by hyperarousal symptoms at 1 month controlling for concurrent psychological distress . Antenatal social support and PTSD symptoms are also included as covariates. Black lines indicate significant pathways, grey lines indicate non-significant pathways. Standardised coefficients and 95% confidence intervals are reported.^*^p < .05; ^**^p < .01; ^***^p <.001. Accounted for 28% of the variance on father-infant bonding.


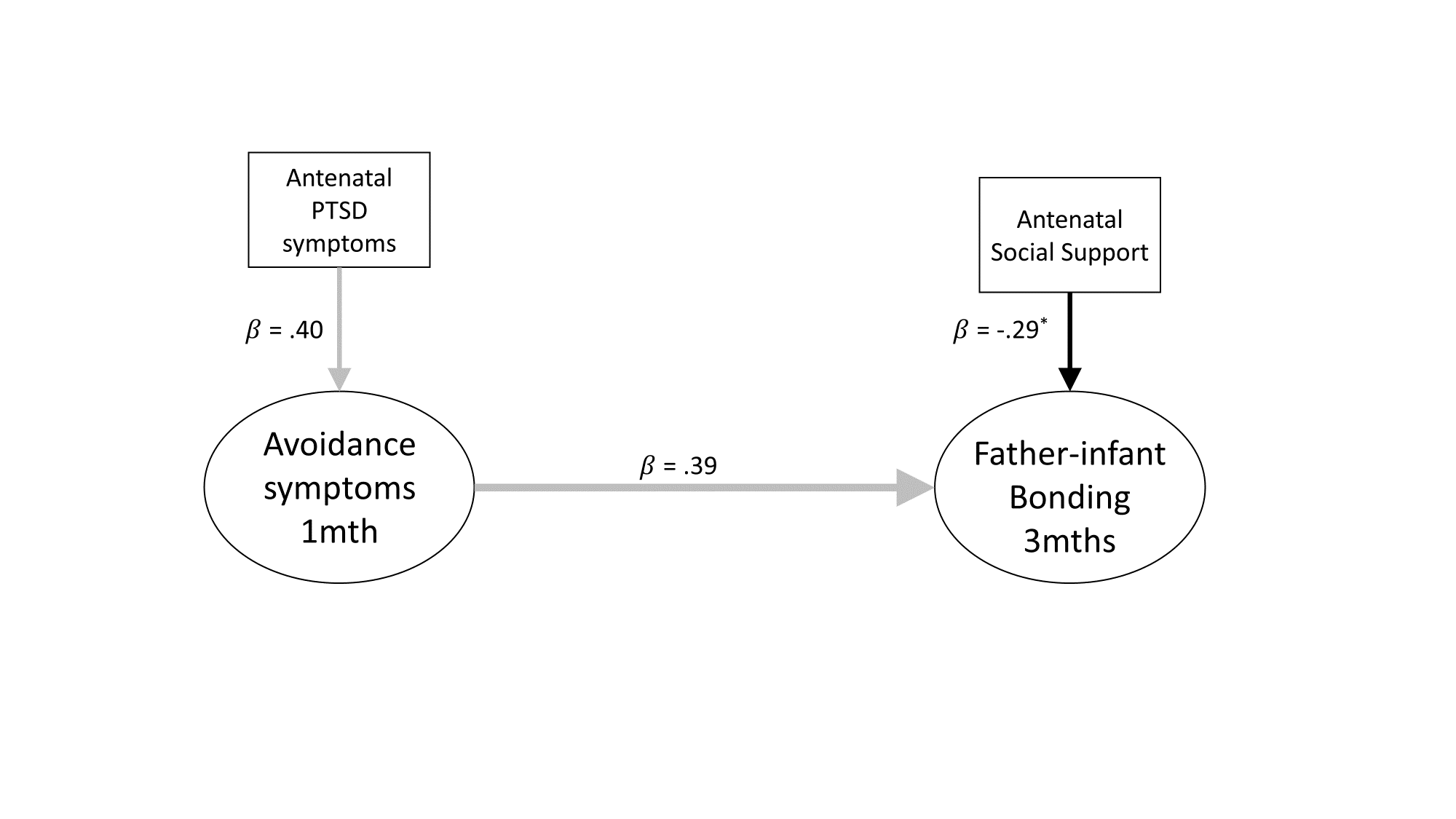


Figure 12. Path model of the prospective prediction of **father**-infant bonding at 3 months by avoidance symptoms at 1 month. Antenatal social support and PTSD symptoms are included as covariates. Black lines indicate significant pathways, grey lines indicate non-significant pathways. Standardised coefficients and 95% confidence intervals are reported^*^p < .05; ^**^p < .01; ^***^p <.001. Accounted for 26% of the variance on father-infant bonding.


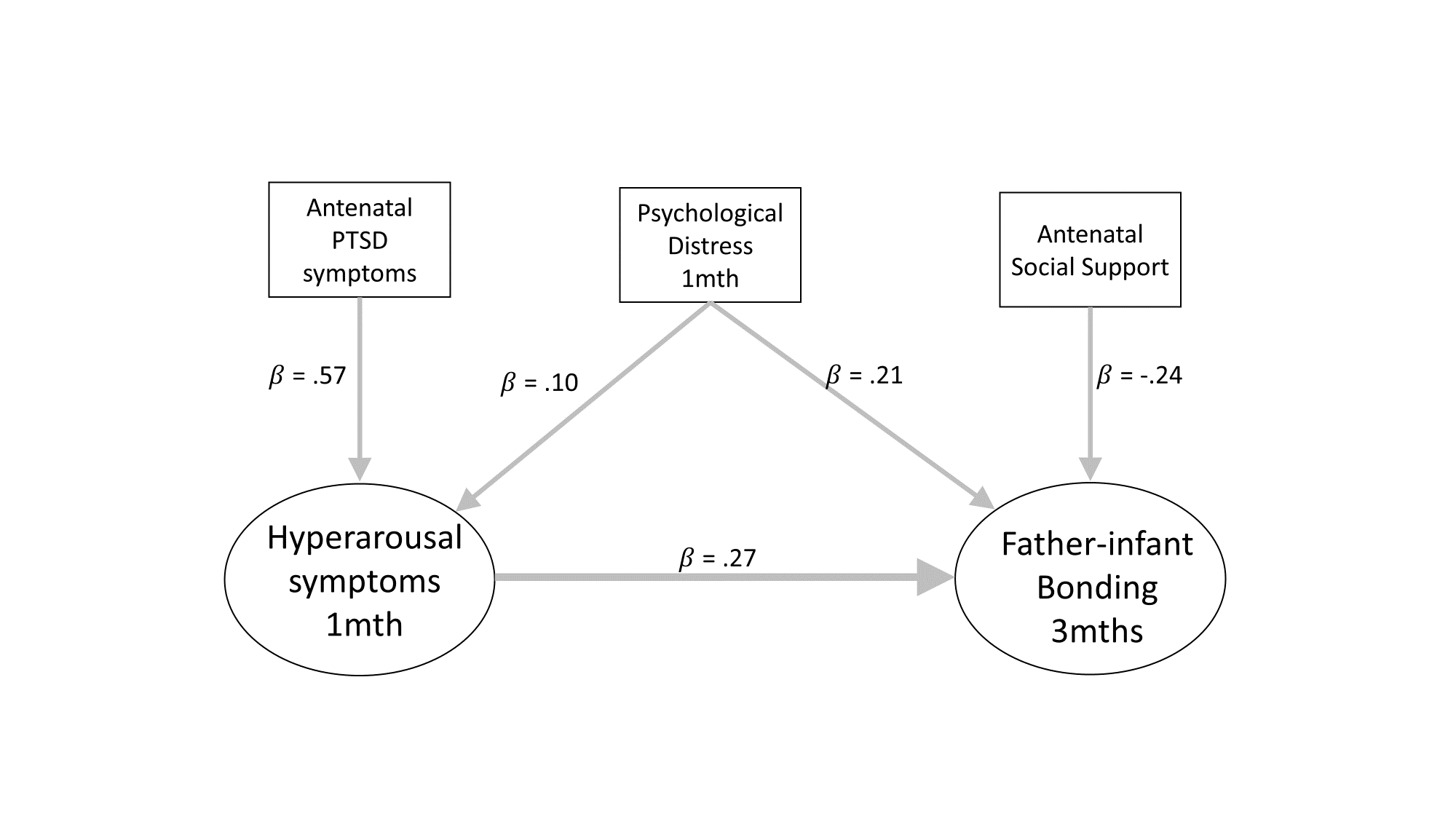


Figure 13. Path model of the prospective prediction of **father**-infant bonding at 3 months by avoidance symptoms at 1 month controlling for concurrent psychological distress . Antenatal social support and PTSD symptoms are also included as covariates. Black lines indicate significant pathways, grey lines indicate non-significant pathways. Standardised coefficients and 95% confidence intervals are reported. ^*^p < .05; ^**^p < .01; ^***^p <.001. Accounted for 31% of the variance on father-infant bonding.
